# ECG Restitution Analysis and Machine Learning to Detect Paroxysmal Atrial Fibrillation: Insight from the Equine Athlete as a Model for Human Athletes

**DOI:** 10.1093/function/zqaa031

**Published:** 2020-11-18

**Authors:** Ying H Huang, Vadim Alexeenko, Gary Tse, Christopher L-H Huang, Celia M Marr, Kamalan Jeevaratnam

**Affiliations:** 1 Faculty of Health and Medical Sciences, University of Surrey, Guildford, GU2 7AL, UK; 2 Tianjin Key Laboratory of Ionic-Molecular Function of Cardiovascular Disease, Department of Cardiology, Tianjin Institute of Cardiology, the Second Hospital of Tianjin Medical University, Tianjin, China; 3 Physiological Laboratory, University of Cambridge, Cambridge, CB2 1QW, UK; 4 Rossdales Equine Hospital and Diagnostic Centre, Exning, CB8 7NN, Suffolk, UK

**Keywords:** diagnostic, ECG, equine athletes, machine learning, paroxysmal atrial fibrillation, restitution analysis

## Abstract

Atrial fibrillation is the most frequent arrhythmia in both equine and human athletes. Currently, this condition is diagnosed via electrocardiogram (ECG) monitoring which lacks sensitivity in about half of cases when it presents in paroxysmal form. We investigated whether the arrhythmogenic substrate present between the episodes of paroxysmal atrial fibrillation (PAF) can be detected using restitution analysis of normal sinus-rhythm ECGs. In this work, ECG recordings were obtained during routine clinical work from control and horses with PAF. The extracted QT, TQ, and RR intervals were used for ECG restitution analysis. The restitution data were trained and tested using *k*-nearest neighbor (*k*-NN) algorithm with various values of neighbors *k* to derive a discrimination tool. A combination of QT, RR, and TQ intervals was used to analyze the relationship between these intervals and their effects on PAF. A simple majority vote on individual record (one beat) classifications was used to determine the final classification. The *k*-NN classifiers using two-interval measures were able to predict the diagnosis of PAF with area under the receiving operating characteristic curve close to 0.8 (RR, TQ with *k *≥* *9) and 0.9 (RR, QT with *k *≥* *21 or TQ, QT with *k *≥* *25). By simultaneously using all three intervals for each beat and a majority vote, mean area under the curves of 0.9 were obtained for all tested *k*-values (3–41). We concluded that 3D ECG restitution analysis can potentially be used as a metric of an automated method for screening of PAF.

## Graphical Abstract

**Figure:**
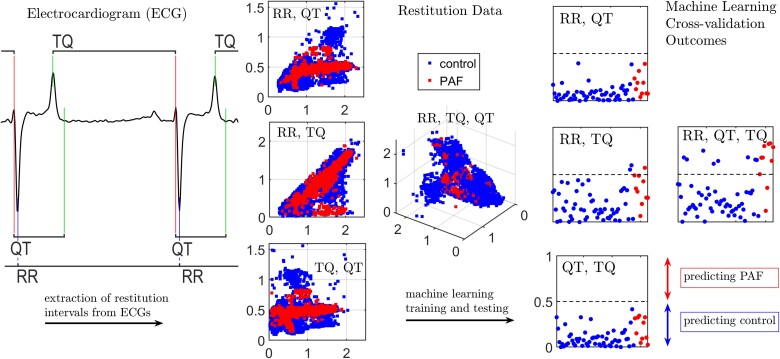

## Introduction

It is well-established that high-performance human athletes are predisposed to cardiac rhythm abnormalities. The same relationship between exercise and cardiovascular disease could be observed in other species, and the most frequent arrhythmia in both equine and human athletes is atrial fibrillation (AF). This condition poses little immediate danger, but negatively affects cardiac function and therefore decreases the athletic performance; it also substantially increases the long-term risk of ischemic events, including stroke.^1^ Although moderate exercise decreases the risks associated with cardiovascular disease, strenuous exercise paradoxically increases arrhythmia risk, including the risk of ventricular arrhythmias that can lead to sudden cardiac death (SCD).^2^ According to the “International recommendations for electrocardiographic interpretation in athletes,”^3^ detection of AF is a “red flag” case when further cardiovascular evaluation is required to investigate for pathologic cardiovascular disorders associated with SCD in athletes. Since equine athletes have a pattern of exercise which is analogous to human athletes and the cardiovascular risks in both species are similar, racehorses might be considered as a feasible model animal to develop the methods of AF detection.

Development of automated methods to detect AF in horses has several advantages. It is a widely occurring clinical problem in veterinary medicine for which there is a growing interest in developing automated detection methods. Additionally, although other large animals have been used as the models for AF research,^4^^,^^5^ we expect that racing horse models offer significant advantages of subjects being kept in tightly controlled environment, with controlled breeding facilitating genetic analysis. Horses also spontaneously developed AF similar to humans. Furthermore, it is routine clinical practice to acquire long durations of equine ECG which makes it easier to develop the algorithms for nonlinear analysis which could be then applied to humans.

Identification of an ongoing AF by ECG analysis is straightforward and easy; however, in nearly half of AF cases which present as paroxysmal AF (PAF), episodes could be short (from hours down to 30 s) and interspersed by very long periods of normal sinus rhythm (see Figure S1 for sample ECG traces). The difficulty of PAF detection prompted the search for other risk markers, based on blood proteins or miRNA, echocardiography, and biomarkers.^6^ However, the current “gold standard” remains the ECG monitoring. Various strategies for “fishing”^7^^,^^8^ for fibrillation episodes were suggested but the most frequent ones use long-term continuous ECG monitoring that requires patient compliance which could be problematic if the recording lasts longer than 2–3 weeks.^9^

There is a growing body of evidence that structural and electrophysiological alterations in the heart associated with the onset of AF might be detectable even in absence of actual AF event and in apparently normal heart. A recent work by Attia et al.^10^ has demonstrated the applicability of neural network-based machine learning techniques in detection of PAF from short strips of normal sinus rhythm ECGs. The “black box” nature of neural network makes it very difficult to make any physiological inference on the nature of ECG alterations which might underlie the detection by the artificial intelligence algorithms. Our recent research has highlighted the feasibility of nonlinear data analysis of equine ECGs for detection of the arrhythmogenic substrate in horses that are in normal sinus rhythm.^11^

One of such techniques that has a straightforward physiological basis is ECG restitution analysis which evaluates the ability of the heart to recover after the heartbeat. It is regarded as an appropriate measure to estimate the integrated mechanoelectrical impact on the heart. There are several possible ways to quantify the ability of the heart to recover after the heartbeat, for example by evaluating QT and TQ interval ratios to compare working phase and resting phase durations. Other estimates include QT and RR ratio, TQ and RR ratio and more complicated ones which include e.g. QRS complex width.^11^ As the physiological changes leading to AF are not confined to atria,^12^ it could be expected that alterations in the key temporal relationships used in restitution analysis, measured from easily resolvable and consistent features of the ventricular ECG will change systematically with rate and rhythm, and some of these can be selected as a basis for indirect detection and related prediction of atrial rhythm disturbances. The common feature of all these analyses is that they rely on interpretation of 2D plots which can be easily performed by a human observer. Unfortunately, this task is not always easy to formalize especially if the analysis concentrates on the outlier detection^13^ and development of appropriate algorithms might be difficult.

**Figure 1.**
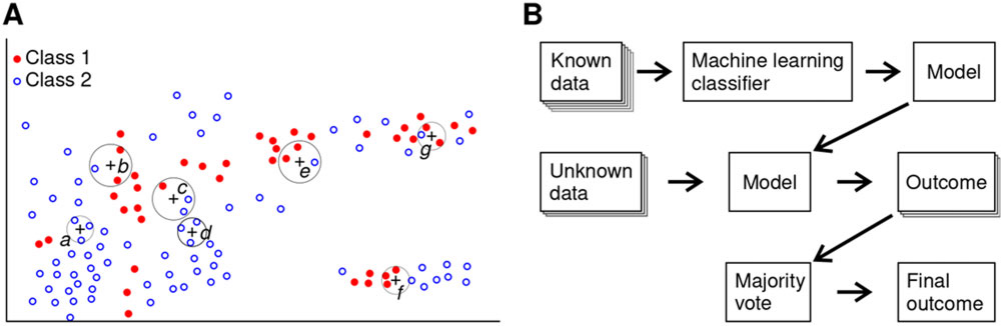
Principles of ECG restitution analysis using k-nearest neighbors classifier machine learning technique. (**A**) Schematic of data classification using the *k*-NN classifier with *k*=3. Data points (crosses) are classified as either in Class 1 or Class 2 based on their *k* closest neighbors (*a,c,d*—Class 1, *b,e,f,g—*Class 2). (**B**) Data flow for the patient classification using the *k*-NN classifier. Extensive preclassified training set (red and blue dots) is used to create the model. Query points from the patient’s records (crosses) are classified individually by classification of three neared neighbors and the outcome for each is decided by a simple majority vote. The final outcome is then decided by subsequent majority vote on all records for that patient.

The task of multidimensional data interpretation has been facilitated in the recent decade by development of high-performance computers and machine learning techniques. In machine learning, the model describing the data is generated not by a human, but rather by a computer algorithm. A popular class of these algorithms is “supervised learning”, which infer the mapping of the inputs to outputs in the “testing” data set using the known relationship of inputs and outputs in a “learning” data set. Of these, a very popular choice is a *k*-nearest neighbors algorithm (*k*-NN), due to its simplicity which ensures easy implementation and excellent interpretability of the results.^14^ During the training phase of the model using *k*-NN classifier, the “training” data are stored in the model. These stored data are then used to classify the new query points by picking *k* nearest neighboring points, based on a pre-defined distance metric and a simple majority vote is carried out (Figure 1). Note that the parameter *k* is often chosen as an odd number due to the nature of majority vote.

A notable disadvantage of restitution analysis is that it typically requires collection and analysis of ECGs recorded at high heart rates, above 110 bpm in horses.^11^ This requires exercising the animal and demands manual examination of the ECG to obtain the cardinal points of the ECG heartbeat waveforms thus preventing the automatization of the whole process. Our previous study highlighted the feasibility of ECG complexity analysis to detect arrhythmogenic substrate, even when ECG recordings were obtained a low heart rate (25–60 bpm) and in normal sinus rhythm. This study necessitated the development of an algorithm to parse (annotate) the ECG signal to detect the QT, TQ, and RR intervals and output them in a format suitable for ECG restitution analysis. Here we investigate the hypothesis that proarrhythmic background present between fibrillation episodes in paroxysmal AF might be detected by restitution analysis of apparently normal sinus-rhythm ECGs in low heart rates, employing the *k*-NN classifier to interpret the outcome of restitution analysis.

## Materials and Methods

### Subject Recruitment

This study did not require an ethical review and received appropriate faculty level approval based on the ethical assessment review checklist by the Non-ASPA Sub-Committee at the University of Surrey. Two-subject cohorts were recruited from Thoroughbred horses of racing age and in race training presented for routine clinical work at Rossdales Equine Hospital and Diagnostic Centre (Newmarket, Suffolk, UK). The control group consisted of 55 healthy horses not displaying clinically significant cardiac abnormalities. The PAF group consisted of 10 horses for which the diagnosis of PAF has been confirmed by ECG analysis. The ECG strips used in this study were obtained during the routine clinical examinations of these horses. These data were previously used to determine the feasibility of complexity analysis of equine telemetric ECG^15^ and to establish the link between heart rate and ECG complexity, as well as to establish the link between ECG complexity and PAF diagnosis.^16^

### Data Recording

Continuous telemetric ECG traces were recorded using a TeleVet 100 recorder (Engel Engineering Services GmbH, Germany), which has signal bandwidth of 0.05–100 Hz and sampling rate of 500 Hz. ECGs were primarily recorded at rest; however, these recordings included a range of heart rates as horses respond to their environment. The duration of recorded ECGs ranged from approximately 15 min to 51 h, with median duration of 83 min.

### Data Preparation

The original data files were exported as comma-separated value (CSV) text files using the built-in function of TeleVet ECG software version 7.0.0. The exported files were plotted using a custom R^17^ script and locations of artifact-free 60-s segments were recorded by a human evaluator. Among others, exclusion criteria included atrial flutter, rapid changes in the heart rate, bigeminy, abrupt baseline alterations, tremors, and identifiable 50 Hz interference to prevent any confounding factors in the analyzed strips. Another R script was used to extract the lead II signal (most commonly used lead to generate rhythm strips^18^) from the accepted segments, resampled to 125 Hz sampling rate and filter using *filtfilt* function. A zero-phase shift digital low-pass fourth-order Butterworth filter was employed with widely used cut-off frequency of 40 Hz.^19^

### ECG Restitution Analysis

**Figure 2.**
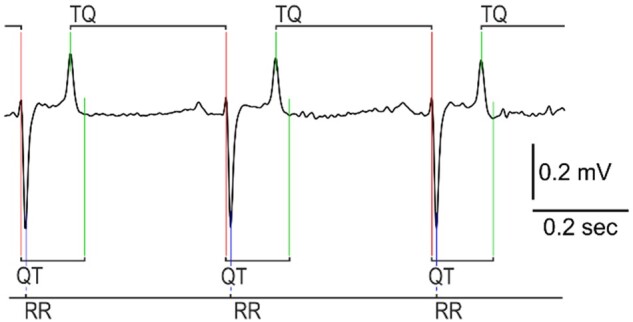
Determination of ECG cardinal points and intervals for restitution analysis. Detected are the Q, R, and T peaks and termination of T wave. Note that while QT interval is defined as the interval between Q peak and termination of T wave, TQ interval is defined as the interval between T and Q peaks.

ECG strips with heart rates greater than 25 bpm were processed by a custom ECG parsing algorithm written in C++, which detected the peak of the Q, R, and T waves, and termination of T wave in each heartbeat waveform (Figure 2) to measure the RR, TQ, and QT intervals. The data for each heartbeat were exported to a CSV file. To eliminate the artifactual detections, only heartbeats where RR and TQ intervals shorter than 2.4 s (corresponding to heart rates of ≥25 bpm) were considered.

The ECG annotation method focused on simultaneous analysis of first derivative of the voltage (dV) and the voltage itself. Briefly, the location of R peak onset was detected as a location where a sequence of 5-samples with low dV (less than 0.1 × SD of dV in the entire signal) was followed by 5 samples of rapidly increased dV (more than 0.2 × SD of dV in the entire signal). The exact location of R peak was refined by a peak search algorithm exploring the area following the detected R wave onset. Then, the signal in the area starting at one-eighth and ending at half of heartbeat duration after R peak was scanned by peak detection algorithm for the T wave peak. The termination of T wave was detected by the decrease of dV below the threshold value (based on the SD of the voltage in the ECG signal). Finally, location of Q peak was detected by peak finding algorithm analyzing the area preceding the R peak. This technique, although crude, was found to be adequate to analyze the high-quality low heart rate equine ECG sampled at 125 Hz, with any abnormalities in the heart rhythm being excluded.

### Patient Classification Using Machine Learning

To quantify the results of restitution analysis, we submitted the detected intervals to *k*-NN classifier algorithm *fitcknn* implemented in MATLAB (Mathworks, USA). Due to low prevalence of PAF in horses,^20^^,^^21^ the PAF cohort was underrepresented compared to controls. As such imbalance in data could significantly impact the performance of machine learning classifier,^22^ we chose to oversample the minority class to match with the majority class, using synthetic minority oversampling technique (SMOTE).^23^ This technique oversamples the minority class by generating synthetic points on straight lines between neighboring points.

Lastly, a simple majority vote is performed on the model predictions of all records (beats) for each subject to determine the final classification of that subject. This step improves the classification performance by combining the predictions using the restitution data derived from the individual heartbeats, so the final predicted classification of a subject is decided by the most predicted classification from all its individual record predictions.

### Cross-Validation for Machine Learning Model

To assess the performance of the model produced by the *k*-NN classifier, we performed a repeated random sampling cross-validation with stratification.^24^ This technique splits the whole data into training and test sets by randomly choosing a subset of subjects for each set. Here the training and validation processes were repeated for different combination of datasets. In each such combination, 48 control horses and 7 PAF horses were selected for a training set and the remaining 7 control and 3 PAF subjects were used as a test set. The process was repeated 1000 times with unique combination of subjects in each run, so the average performance of the model when dealing with unknown data can be estimated.

## Results

### ECG Restitution Analysis

**Figure 3.**
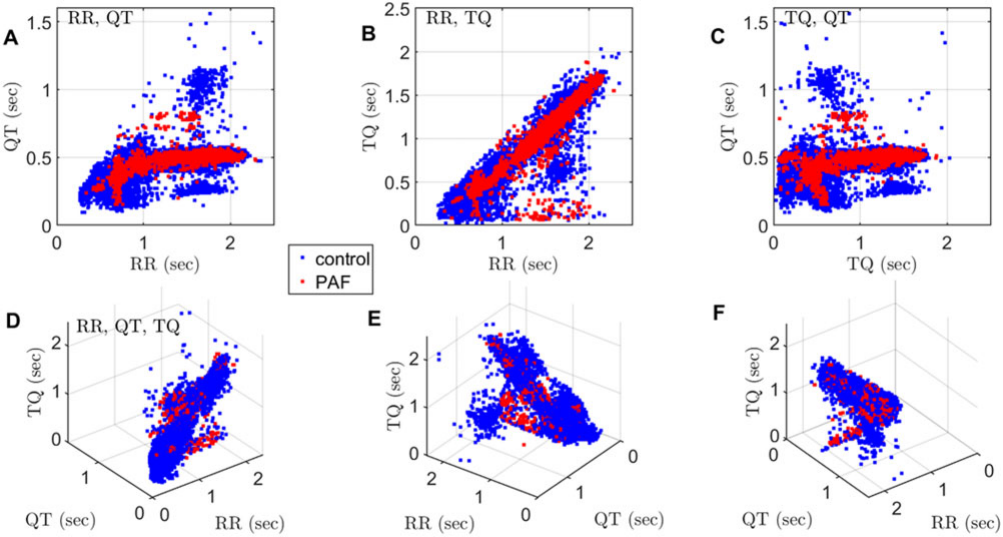
ECG restitution analysis using two and three parameters simultaneously. Each dot represents an individual heartbeat; blue for controls and red for cases (PAF). The total number of analyzed heartbeats is 43* *331 (34* *861 from control and 8470 from PAF). (**A**) Dependence of the action potential duration (represented by QT interval) on the basic cycle length (RR interval). (**B**) Dependence of the diastolic interval (represented by TQ interval) on the basic cycle length (RR). (**C**) Dependence on the action potential duration on the diastolic interval. (**D–F**) Snapshots of data at different angles reveal a complex interdependence between all three parameters used for restitution analysis.

To estimate the link between arrhythmogenic substrate and PAF we performed three analyses of relationships: between QT and RR intervals, TQ and RR intervals, and TQ and QT intervals (Figure 3A–C). We observed that the distribution of data points is similar in both cohorts however the distribution patterns were slightly different in cases and controls. Given that RR, TQ, and QT intervals for each heartbeat are strongly correlated, we decided to evaluate whether the spatial distribution of data points might provide an additional insight. We thus plotted the QT, TQ, RR intervals in a 3D space (Figure 3D–E). It became apparent that most data point clusters around an inclined plane. Traditionally restitution analysis is performed using 2D relationships^11^ using either QT and RR, or TQ and RR, or QT and TQ interval pairs. However, here we identify the relationship is in fact 3D; thus, analysis using 2D relationships might be suboptimal in estimating the differences between PAF and control.

### Application of k-Nearest Neighbor Algorithm to the Restitution Data

The characteristic distribution of data points on the 2D and 3D graphs suggested the possibility that diagnosis of PAF might be associated with specific pattern of distribution of the data. To quantify this association, we used models built using the *k*-NN machine learning algorithm, to analyze the QT and RR, TQ and RR, QT and TQ interval pairs as well as QT, TQ, and RR intervals all together.

To assess the overall performance of the models produced by the *k*-NN classifier on different combinations of restitution intervals, we performed the stratified repeated random sampling cross-validation technique as previously described, with 1000 runs for consecutive odd values of neighbors *k* ranging from 3 to 41. Small *k*-values tend to produce complex models which overfit to the data and they are more susceptible to noise (high variance, low bias), while large *k* values tend to produce simple models that are typically unaffected by noise but may fail to capture the true boundaries between different classes (low variance, high bias) and it is more computationally expensive. Therefore, it is important to identify the optimal *k* value for the *k*-NN classifier. The distance function of the *k*-NN classifier was chosen to be the standardized Euclidean distance, and we also normalized the data intervals (RR, QT, TQ) by their means and SDs to eliminate any influence caused by magnitude differences between the restitution intervals.

**Figure 4.**
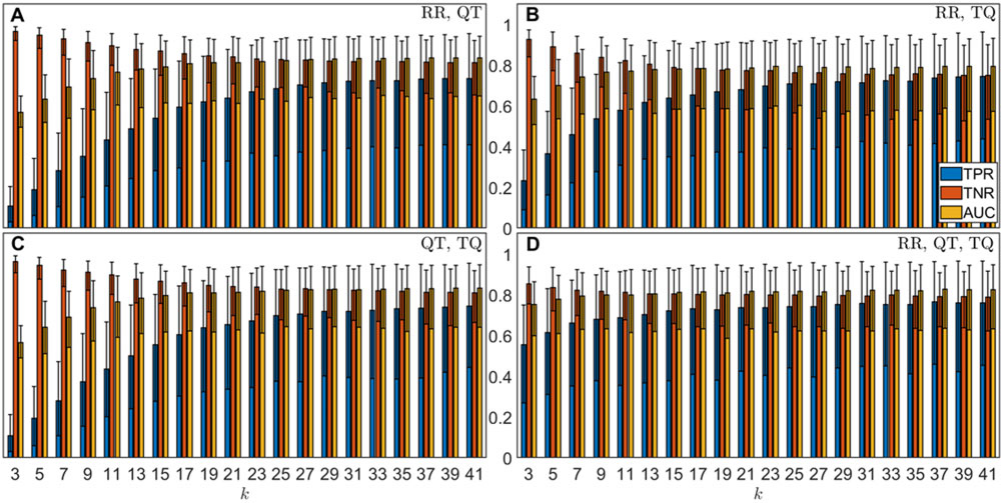
Results of model for classifying individual records, over 1000 repeated random cross-validation runs. Each run uses randomly selected 48 controls and 7 PAF horses for training set and 7 controls and 3 PAF horses for validation set out of 55 controls and 10 cases. Blue bars show the mean TPRs; red bars show the mean TNRs; yellow bars show the mean AUCs. The error bars show the corresponding 2.5th and 97.5th percentile of the metrics.

The receiver operating characteristic (ROC) curve provides the sensitivity and specificity of a model at different classification thresholds, and its area under the curve (AUC) measures the overall predictive power of the model.^25^ In this case, the models produced mean AUCs of up to 0.836 + 0.107 − 0.188 (RR, QT), 0.795 + 0.137 − 0.221 (RR, TQ), and 0.832 + 0.113 − 0.193 (QT, TQ) within the 2.5th and 97.5th percentiles of the data. For these cases, higher mean AUCs were obtained at large *k*-values with the highest values achieved at *k *=* *41. Using all three intervals (RR, TQ, QT), the machine learning model gave rise to mean AUC of up to 0.827 + 0.114 − 0.192 which is similar to the values obtained using only two intervals, however, there are less variations in the mean AUC values and other metrics across different *k* values (see Tables S1–S4). By choosing PAF as the positive class, this is also reflected in Figure 4 where we observed much more dramatic increases in the mean true positive rates (TPRs) of up to around 0.74 with rising *k* values for the model predictions that used only two intervals, whereas the mean TPR (of up to around 0.76) stabilizes very quickly for the model prediction using all three intervals and the changes in the mean AUCs are also smaller as previously mentioned. The TPR is an important metric that measures the PAF detection ability of the model, especially since our data are imbalanced. Additionally, decreases in the mean true negative rates (TNRs) indicate the models become less specific as *k* increases, as shown in Figure 4. The ROC curves for specific values of *k* (*k *=* *5, 13, 23, 37) can be found in Figures S2–S3 where we observed a better performance for model using all three intervals compared to those using only two intervals, especially for small *k* values.

The final scores produced by a machine learning model are the posterior probabilities of the records, which correspond the likelihood of each record being PAF, with values ranging from 0 (control) to 1 (PAF). The classification threshold is unknown as it is determined by the internal *k*-NN algorithm. The posterior probability produced by the machine learning model for specific values of *k* (*k *=* *5, 13, 23, 37) are shown in Figures S4–S5 where we observed higher sensitivity (higher posterior probabilities for most records) for large *k*-values, and as expected this change is more prominent for models using two intervals. For *k *=* *5, we see the models cannot distinguish between control and PAF records by using only 2 out of 3 interval measures since the corresponding plots in Figure S4 shows that the posterior probabilities of the control and PAF have similar distributions, whereas the posterior probability distribution of PAF is weighted more heavily toward 1 compared to control for classification using all 3 interval measures as shown in the top right image (with RR, QT, TQ) of Figure S4.

### Majority Vote on Individual Record Classification

**Figure 5.**
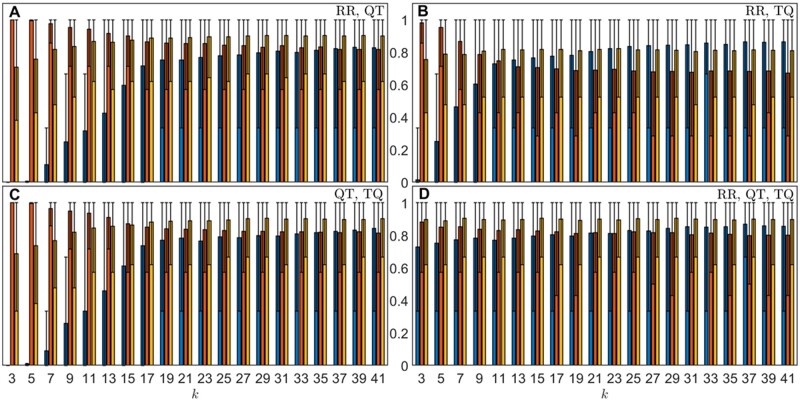
Results using majority votes on all records in each animal, over 1000 repeated random cross-validation runs. Each run uses randomly selected 48 controls and 7 PAF horses for training set and 7 controls and 3 PAF horses for validation set out of 55 controls and 10 cases. Blue bars show the mean TPRs; red bars show the mean TNRs; yellow bars show the mean AUCs. The error bars show the corresponding 2.5th and 97.5th percentile of the metrics.

**Figure 6.**
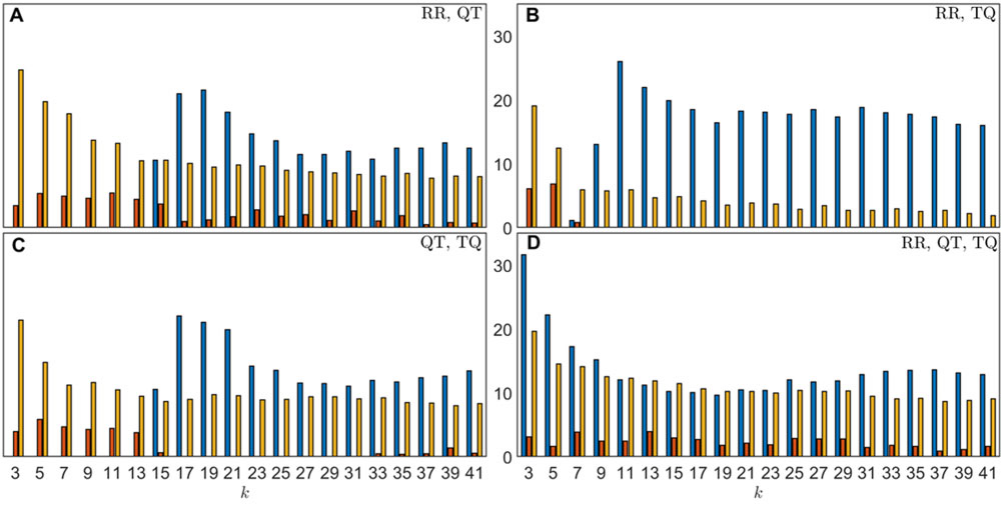
The percentage increases from using majority votes on all records in each animal, over 1000 repeated random cross-validation runs. Each run uses randomly selected 48 controls and 7 PAF horses for training set and 7 controls and 3 PAF horses for validation set out of 55 controls and 10 cases. Blue bars show the percentage increase in mean TPRs; red bars show the percentage increase in mean TNRs; yellow bars show the percentage increase in mean AUCs.

As means to improve the analysis outcome, we performed the majority vote on all patient’s records to make a final classification on the subject. Results using majority vote (Tables S5–S8) were found to have noticeably better performance. Although the performances in the mean TPRs are worse for models using two intervals at low *k* values (*k* < ∼13), nearly all metrics are more consistent and stay the same levels using all three intervals compared the corresponding record classification as shown in Figure 5. For model with all three intervals, the mean TPRs, TNR, and AUCs have all increased by around 10%–31%, 1%–4%, and 9%–20%, respectively, for different *k*-values using majority votes (see Figure 6 for detail). Note that the large uncertainties in metrics obtained using majority votes are due to the small test size of the data (7 control and 3 PAF subjects), and they are expected to decrease when more data become available which allow for a larger test data size.

**Figure 7.**
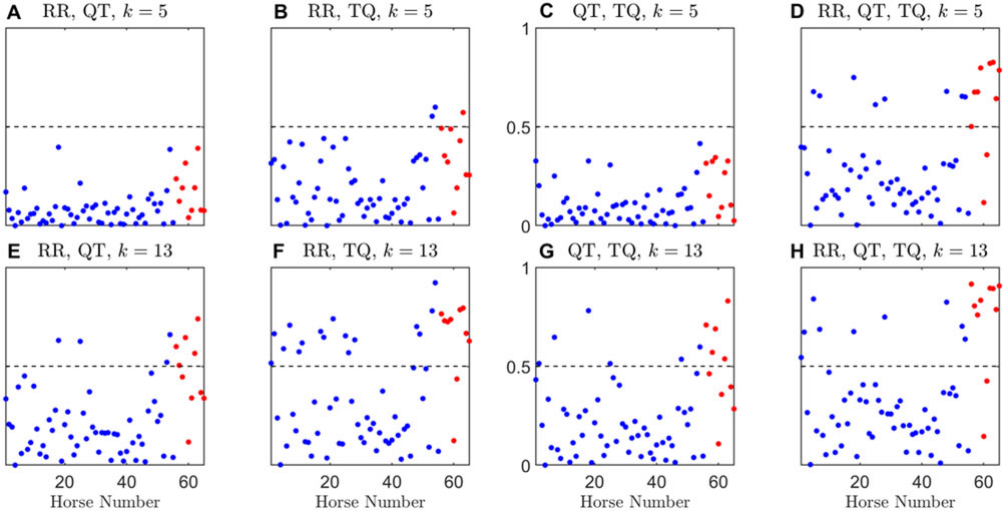
Posterior probability of using majority votes on all records in each animal, over 1000 repeated random cross-validation runs, for *k* = 5, 13. Subjects belong to the control class are denoted by blue solid dots and PAF class by red solid dots. Results were averaged over 1000 random cross-validation runs. Dashed line indicates the majority vote threshold which is at 0.5.

**Figure 8.**
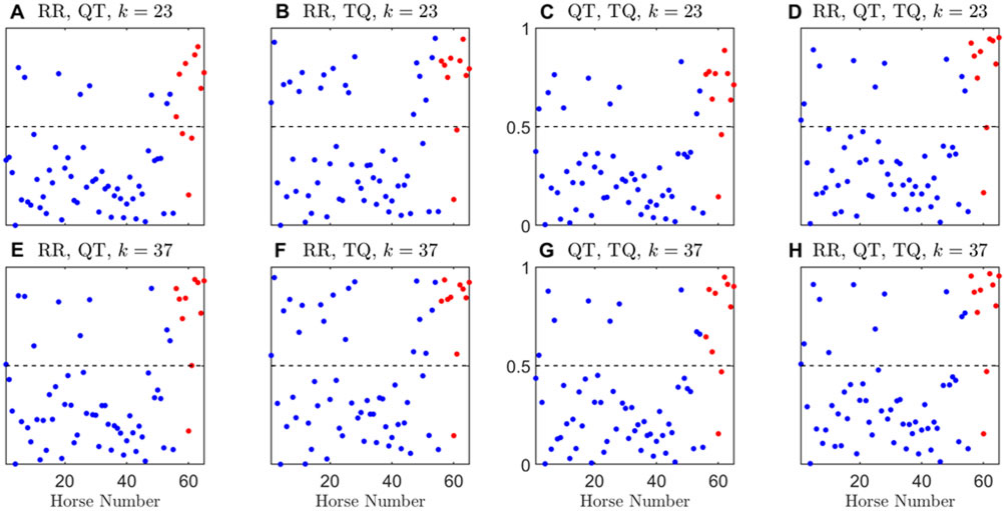
Posterior probability of using majority votes on all records in each animal, over 1000 repeated random cross-validation runs, for *k* = 23, 37. Subjects belong to the control class are denoted by blue solid dots and PAF class by red solid dots. Results were averaged over 1000 random cross-validation runs. Dashed line indicates the majority vote threshold which is at 0.5.

The posterior probability threshold in the case of majority vote was chosen to be 0.5 since it is a simple majority vote of the individual records. Figures 7–8 show the posterior probability by majority votes of each animal for *k *=* *5, 13, 23, 37. We observed that the models with only two combinations of the interval measures cannot distinguish between control and PAF at low *k*-values (e.g., *k *=* *5) and they can barely distinguish the two classes at *k *=* *13, whereas the performance of the model with all three interval measures is more consistent for all the chosen *k*-values, which shows a similar pattern to the individual record classification results from the previous section.

The differences between the metrics for the individual records and the metrics obtained using majority votes of the records are significant, especially for the mean TPRs and AUCs, with their, respectively, *P*-values being near zero (less than 10^−64^ and 10^−107^, respectively) using the Wilcoxon signed-rank test^26^ (see Tables S9–S12) as the two sets of metrics are related (paired) and non-Gaussian. For models with two intervals, there are increases for the mean TPRs for *k *>* *13, and increases in the mean AUCs for all tested *k*-values with significant differences (*P*-values less than 10^−98^ for RR, QT; 10^−8^ for RR, TQ; and 10^−63^ for QT, TQ) which indicates the overall predictive powers of the models have indeed increased using majority votes.

## Discussion

During recent years, a strong interest has emerged in detection of PAF by nonlinear analysis of normal sinus rhythm ECG. This research was facilitated by the appearance of freely accessible public ECG databases and massive increase in processing power available for biomedical research. One notable example is Physionet^27^ which contains a large collection of open-access datasets of physiological signals including ECG recordings for a variety of conditions. Together with the emergence of new artificial intelligence technologies, this suggested that an acute problem of developing a reliable biomarker for PAF could potentially be solved using an inexpensive technique that can be easily automated. In particular, this has led to several studies in the detection of various arrhythmia conditions including AF using machine learning in recent years. Many machine learning methods have been proposed for classifying various arrhythmia conditions using ECG features with great success by achieving extremely high accuracies.^28–31^ However, the problem of detecting condition using only normal sinus rhythm signals is much harder. One recent study on detection of AF in human using normal sinus rhythm ECGs has achieved good result with an AUC of 0.87 (95% CI 0.86–0.88).^10^ Their study involves more than 180* *000 patients with multiple ECGs recorded for longer than a month, and they use deep learning technique for the classification, which requires a vast amount of data. Our approach has the advantage of using far less subjects with significantly shorter ECGs, and we achieved similar result (see Table S8).

Our study highlights the feasibility of equine model to study the mechanisms of spontaneously developing PAF. Widely used in cardiovascular research rodents have typical heart rate range of 310–840 bpm for mice or 250–490 bpm for rats,^32^ which is very different for the human heart rate range. Typical resting heart for adult horses range between 32 and 38 bpm^33^ and can peak up to around 240 bpm^34^ at high exercise intensity rate. Human athletes have been previously reported to have a “lowest resting heart rate” of between 24 and 48 bpm.^35^ A healthy adult human depending on age (e.g., 20–60 years old) may have a maximal heart rate of between 160–200 bpm based on established calculations.^36^ These data suggest the horses and human have much closer comparable heart rate ranges.

We have previously demonstrated that complexity analysis of low heart rate ECG might be indicative of PAF in horses.^16^ Our current finding suggests that not only complexity analysis, which analyses the changes of the information content in ECG, determined by changes in the heartbeat waveforms resulting from the pathological alterations in the myocardium, but also ECG restitution analysis, which analyses the ability of the heart to recover after the heartbeat can reveal proarrhythmic background associated with PAF. Since complexity analysis evaluates different aspects of a signal compared to restitution analysis it could be speculated that a combination of such biomarkers might provide additional increase in performance in detection of this condition. One may also expect that the future prospective studies might consider other factors, for example, basic clinical data (age, sex, weight, blood pressure), or any blood-based biomarkers^6^ as additional prognostic factors.

Our study highlights the potential of a simple machine learning algorithm that allows for use of diagnostic markers that are hard to quantify using more conventional techniques, like regression analysis. However, the *k-*NN classifier we use is a computationally intensive technique that compares the distribution of data points in the “unknown” queried data set with a “teaching” data set. This might cause systematic error if the patient data are obtained by a different acquisition hardware, with different sampling rates and different low-pass filter parameters. To facilitate comparison of ECG recordings obtained from different sources, we used strong ECG filtering with low cut-off 40 Hz and resampled the signal to 125 Hz sampling rate. Still, it could be argued that to make the analysis results less dependent on the properties of “teaching” data set other machine learning techniques should be explored in the future studies.

The optimal choice of *k* varies from the combination of the intervals chosen, however, the variations in the performance metrics caused by different values of *k* are much smaller for model with all three interval measures compared to those using only two intervals. This is important since the inclusion of more data to our current model or model trained using different data may have different optimal *k-*values, and a model that is less sensitive to the choice of *k* is clearly more desirable in practice. Additionally, we found models using only two intervals tend to perform worse for small *k* values, whereas model using all three intervals perform much better for small *k-*values which are preferred since *k*-NN can be very computationally expensive for large *k*-values especially if the dataset is large, though the trade-off between large *k* and the additional dimension is unclear at this stage.

A common feature of both complexity and ECG restitution analysis is that they require ECG strips that are substantially longer than the standard ECG strip duration which is currently accepted in the healthcare settings. Currently, a typical 12-lead human ECG recording has about 6–10 s of Lead II data. This would limit the detection ability for many nonlinear analytical methods. The necessity of longer strips (30–120 s) was already highlighted in the literature.^37^ Of course, requiring longer recordings may consume additional clinical time. However, considering the time it takes to setup and places the ECG electrode on patients, an additional 30–120 s would not noticeably increase the time budget for an ECG procedure. Thus, it seems reasonable to expect that going forward it should be standard practice to take longer recordings between 30 and 120 s.

One of the interesting observations of this study is a link between QT, TQ, and RR intervals used in the ECG restitution analysis. While the existence of inter-relationship between these intervals is not surprising and should be considered as trivial, we demonstrate that “traditional” restitution analysis^11^^,^^13^^,^^38–40^ is in fact dealing with projections of the 3D inclined plane defined by the durations of these intervals on the three planes defined by the pairs of QT, TQ, and RR axes. As the inclination of data plane in relation to projection surfaces leads to the compression of distances between data points and shape of the pattern formed by them, it could be argued that the definitive form of ECG restitution analysis should probably consider the distribution of the data points on the inclined surface in the 3D feature space.

Our results suggest that identification of AF in horses, or at least identification of very high-risk groups might possibly be achieved using short ECG recordings. Screening programs to detect asymptomatic disorders have long become the routine for human athletes, and we suggest that such screening could be introduced in veterinary practice. Translation of this study to human medicine might be an interesting endeavor due to the similarity of AF dynamics in both species.^41^

Our study has some limitations. The retrospective nature of this research has limited data availability and we were not able to conduct the extensive validation, resorting instead to bootstrapping techniques. Our equine ECG parsing (annotation) algorithm is intentionally limited to the analysis of the high quality and low heart rate (<90 bpm) recordings, which do not contain any artifacts. Therefore, a robust algorithm has to be created and verified in an appropriate study.

To conclude, while the “classic” ECG restitution analysis considers the combination of two parameters,^11^^,^^39^ we propose a novel method of restitution analysis (which could be defined as 3D ECG restitution analysis or 3D-ERA) which simultaneously uses three easily detectable intervals. It could be expected that the combination 3D-ERA and machine learning might allow detection of PAF in both equine and human patients.

## Supplementary Material

Supplementary material is available at the *APS Function* online.

## Funding

The work was supported by PetPlan Charitable Trust (Guildford, UK) [grant S17-447-485]; and the EPSRC Impact Acceleration Award (London, UK) to the University of Surrey.

## Conflict of Interest Statement

The authors declare no conflict of interest.

## Authors’ Contributions

Y.H.H. and V.A. analyzed data, wrote first draft of manuscript, and reviewed all subsequent draft of manuscripts; G.T. provided technical assistance for analysis and reviewed all drafts of manuscript; C.L.H.-H. secured funding and reviewed all drafts of manuscript; C.M.M. collected data, secured funding, and reviewed all drafts of manuscript; K.J. designed study, secured funding, reviewed all drafts of manuscript.

## Data Availability Statement

The data underlying this article cannot be shared publicly to preserve the identity of the study subjects. The data will be shared on reasonable request to the corresponding author, subject to signing the appropriate NDA. The requests for the executables and source code should be addressed to the corresponding author.

## Supplementary Material

zqaa031_Supplementary_Data

## Notes

Ying H. Huang and Vadim Alexeenko are joint first authors.
