# Supplementary material for: ECG Restitution Analysis and Machine Learning to Detect Paroxysmal Atrial Fibrillation: Insight from the Equine Athlete as a Model for Human Athletes
Source: Function (Oxf). 2020 Nov 18;2(1):zqaa031. doi: 10.1093/function/zqaa031 (PMC8788737; doi:10.1093/function/zqaa031)
Supplement: zqaa031_Supplementary_Data [file zqaa031_supplementary_data.zip › Supplementary figure legends 6 - Ying Edit.docx]

Supplementary figure legends for:

**ECG restitution analysis and machine learning to detect paroxysmal atrial fibrillation: insight from the equine athlete as a model for human athletes**

Short title: **PAF detection using ECG restitution analysis**

Ying H. Huang^1,#^, Vadim Alexeenko^1,#^, Gary Tse^2^, Christopher L.H-Huang^1,3^, Celia M. Marr^4^ and Kamalan Jeevaratnam^1,3,*^

**Supplementary Figure 1.** *Sample ECG from two horses diagnosed with PAF*. In each pair, top ECG demonstrates the heart rhythm during the fibrillation episode and the bottom one the normal sinus rhythm after spontaneous restoration of it. The time interval between the end of the top strip and the beginning of a second one is 60 seconds. The top and bottom pairs correspond to horse numbers 58 and 65 respectively, with returns to normal sinus rhythms occurring after 36 seconds and 42 seconds of the corresponding AF episodes shown above, respectively.

**Supplementary Figure 2.** *ROC curves of model for classifying individual records, over 1000 repeated random cross-validation runs, for k = 5, 13.*

Solid light blue lines show all individual ROC curves over 1000 random cross-validation runs with the solid black line show the corresponding median ROC curve.

**Supplementary Figure 3.** *ROC curves of model for classifying individual records, over 1000 repeated random cross-validation runs, for k = 23, 37.*

Solid light blue lines show all individual ROC curves over 1000 random cross-validation runs with the solid black line show the corresponding median ROC curve.

**Supplementary Figure 4.** *Posterior probability of model for classifying individual records, over 1000 repeated random cross-validation runs, for k =5, 13.*

Records belong to the control class are denoted by blue solid dots and PAF class by red solid dots. Results were averaged over 1000 random cross-validation runs.

**Supplementary Figure 5.** *Posterior probability of model for classifying individual records, over 1000 repeated random cross-validation runs, for k =23, 37.*

Records belong to the control class are denoted by blue solid dots and PAF class by red solid dots. Results were averaged over 1000 random cross-validation runs.
