# Supplementary material for: ECG Restitution Analysis and Machine Learning to Detect Paroxysmal Atrial Fibrillation: Insight from the Equine Athlete as a Model for Human Athletes
Source: Function (Oxf). 2020 Nov 18;2(1):zqaa031. doi: 10.1093/function/zqaa031 (PMC8788737; doi:10.1093/function/zqaa031)
Supplement: zqaa031_Supplementary_Data [file zqaa031_supplementary_data.zip › Supplementary tables.docx]

Supplementary data for:

**ECG restitution analysis and machine learning to detect paroxysmal atrial fibrillation: insight from the equine athlete as a model for human athletes**

Short title: **PAF detection using ECG restitution analysis**

Ying H. Huang^1,#^, Vadim Alexeenko^1,#^, Gary Tse^2^, Christopher L.H-Huang^1,3^, Celia M. Marr^4^ and Kamalan Jeevaratnam^1,3,*^

**Supplemental Table 1**.

Performance metrics of the k-NN classifier by records (PAF as the true class) for the RR, QT intervals with odd values of k ranging from 3 to 41. Results correspond to the means and the 95% coverage intervals of the random cross-validation runs over 1000 runs (- and + correspond to the 2.5th and 97.5th percentiles respectively), for the machine learning model. The displayed metrics are accuracy (ACC), true positive rate (TPR), true negative rate (TNR), area under the receiver operating characteristic curve (AUC), positive predictive value (PPV), negative predictive value (NPV), F_1_ score for PAF subjects (F_1_ PAF) and F_1_ score for control subjects (F_1_ CTR).

| *k* | ACC | TPR | TNR | AUC | PPV | NPV | F_1_ (PAF) | F_1_ (CTR) |
| --- | --- | --- | --- | --- | --- | --- | --- | --- |
| 3 | 0.639 +0.183 -0.209 | 0.107 +0.097 -0.077 | 0.967 +0.024 -0.045 | 0.568 +0.080 -0.071 | 0.646 +0.253 -0.367 | 0.634 +0.197 -0.238 | 0.181 +0.142 -0.125 | 0.760 +0.141 -0.202 |
| 5 | 0.656 +0.171 -0.196 | 0.188 +0.154 -0.128 | 0.947 +0.038 -0.065 | 0.634 +0.120 -0.114 | 0.669 +0.240 -0.329 | 0.647 +0.199 -0.247 | 0.288 +0.191 -0.183 | 0.763 +0.138 -0.212 |
| 7 | 0.688 +0.150 -0.185 | 0.282 +0.186 -0.176 | 0.930 +0.046 -0.081 | 0.694 +0.137 -0.153 | 0.694 +0.204 -0.324 | 0.677 +0.187 -0.242 | 0.394 +0.200 -0.216 | 0.779 +0.128 -0.196 |
| 9 | 0.707 +0.135 -0.184 | 0.353 +0.233 -0.202 | 0.912 +0.054 -0.092 | 0.735 +0.137 -0.155 | 0.690 +0.196 -0.284 | 0.698 +0.172 -0.254 | 0.460 +0.224 -0.225 | 0.787 +0.118 -0.197 |
| 11 | 0.722 +0.124 -0.178 | 0.434 +0.234 -0.227 | 0.895 +0.062 -0.095 | 0.765 +0.122 -0.161 | 0.705 +0.182 -0.260 | 0.716 +0.171 -0.254 | 0.529 +0.204 -0.227 | 0.791 +0.114 -0.193 |
| 13 | 0.734 +0.121 -0.173 | 0.488 +0.247 -0.245 | 0.879 +0.074 -0.102 | 0.780 +0.127 -0.188 | 0.699 +0.185 -0.253 | 0.733 +0.171 -0.256 | 0.566 +0.194 -0.227 | 0.795 +0.112 -0.191 |
| 15 | 0.748 +0.113 -0.173 | 0.540 +0.241 -0.257 | 0.869 +0.080 -0.118 | 0.793 +0.125 -0.177 | 0.707 +0.170 -0.249 | 0.751 +0.167 -0.255 | 0.604 +0.182 -0.219 | 0.802 +0.110 -0.187 |
| 17 | 0.761 +0.104 -0.166 | 0.593 +0.228 -0.298 | 0.857 +0.082 -0.126 | 0.808 +0.117 -0.195 | 0.711 +0.167 -0.256 | 0.770 +0.161 -0.255 | 0.638 +0.174 -0.238 | 0.808 +0.103 -0.186 |
| 19 | 0.765 +0.101 -0.165 | 0.620 +0.224 -0.292 | 0.847 +0.085 -0.133 | 0.812 +0.115 -0.188 | 0.705 +0.167 -0.246 | 0.780 +0.153 -0.253 | 0.652 +0.167 -0.233 | 0.809 +0.100 -0.186 |
| 21 | 0.769 +0.102 -0.173 | 0.638 +0.240 -0.309 | 0.841 +0.099 -0.132 | 0.812 +0.120 -0.200 | 0.703 +0.164 -0.245 | 0.789 +0.155 -0.242 | 0.659 +0.180 -0.225 | 0.810 +0.101 -0.164 |
| 23 | 0.774 +0.100 -0.152 | 0.669 +0.230 -0.302 | 0.832 +0.092 -0.140 | 0.818 +0.116 -0.185 | 0.703 +0.157 -0.254 | 0.800 +0.153 -0.249 | 0.677 +0.165 -0.240 | 0.812 +0.100 -0.186 |
| 25 | 0.778 +0.101 -0.160 | 0.686 +0.230 -0.330 | 0.829 +0.102 -0.138 | 0.823 +0.113 -0.201 | 0.701 +0.171 -0.235 | 0.810 +0.150 -0.249 | 0.683 +0.174 -0.245 | 0.815 +0.103 -0.180 |
| 27 | 0.784 +0.099 -0.141 | 0.703 +0.221 -0.330 | 0.826 +0.098 -0.134 | 0.828 +0.110 -0.186 | 0.704 +0.161 -0.224 | 0.818 +0.145 -0.242 | 0.694 +0.163 -0.233 | 0.818 +0.098 -0.158 |
| 29 | 0.787 +0.096 -0.167 | 0.715 +0.215 -0.335 | 0.822 +0.106 -0.151 | 0.832 +0.109 -0.196 | 0.698 +0.163 -0.230 | 0.829 +0.138 -0.262 | 0.697 +0.165 -0.229 | 0.821 +0.097 -0.180 |
| 31 | 0.788 +0.097 -0.141 | 0.722 +0.218 -0.330 | 0.819 +0.110 -0.153 | 0.833 +0.109 -0.195 | 0.701 +0.167 -0.244 | 0.830 +0.137 -0.258 | 0.702 +0.166 -0.229 | 0.820 +0.096 -0.172 |
| 33 | 0.789 +0.096 -0.141 | 0.723 +0.217 -0.323 | 0.820 +0.106 -0.132 | 0.834 +0.104 -0.184 | 0.702 +0.159 -0.226 | 0.831 +0.136 -0.245 | 0.703 +0.168 -0.237 | 0.822 +0.093 -0.170 |
| 35 | 0.786 +0.102 -0.132 | 0.723 +0.220 -0.328 | 0.817 +0.103 -0.143 | 0.833 +0.108 -0.185 | 0.701 +0.161 -0.235 | 0.829 +0.138 -0.243 | 0.703 +0.165 -0.231 | 0.819 +0.098 -0.154 |
| 37 | 0.788 +0.098 -0.157 | 0.732 +0.216 -0.325 | 0.815 +0.110 -0.154 | 0.835 +0.107 -0.198 | 0.703 +0.163 -0.239 | 0.831 +0.139 -0.253 | 0.708 +0.167 -0.225 | 0.819 +0.101 -0.189 |
| 39 | 0.788 +0.098 -0.159 | 0.735 +0.216 -0.325 | 0.812 +0.114 -0.146 | 0.836 +0.105 -0.191 | 0.700 +0.163 -0.215 | 0.832 +0.139 -0.270 | 0.708 +0.165 -0.231 | 0.817 +0.100 -0.182 |
| 41 | 0.789 +0.098 -0.145 | 0.736 +0.221 -0.325 | 0.812 +0.110 -0.159 | 0.836 +0.107 -0.188 | 0.700 +0.168 -0.245 | 0.834 +0.137 -0.269 | 0.709 +0.168 -0.240 | 0.819 +0.097 -0.179 |

**Supplemental Table 2**.

Performance metrics of the k-NN classifier by records (PAF as the true class) for the RR, TQ intervals with odd values of k ranging from 3 to 41. Results correspond to the means and the 95% coverage intervals of the random cross-validation runs over 1000 runs (- and + correspond to the 2.5th and 97.5th percentiles respectively), for the machine learning model. The displayed metrics are accuracy (ACC), true positive rate (TPR), true negative rate (TNR), area under the receiver operating characteristic curve (AUC), positive predictive value (PPV), negative predictive value (NPV), F_1_ score for PAF subjects (F_1_ PAF) and F_1_ score for control subjects (F_1_ CTR).

| *k* | ACC | TPR | TNR | AUC | PPV | NPV | F_1_ (PAF) | F_1_ (CTR) |
| --- | --- | --- | --- | --- | --- | --- | --- | --- |
| 3 | 0.665 +0.156 -0.196 | 0.233 +0.150 -0.143 | 0.926 +0.048 -0.086 | 0.634 +0.110 -0.126 | 0.649 +0.215 -0.306 | 0.660 +0.186 -0.241 | 0.338 +0.164 -0.191 | 0.766 +0.130 -0.202 |
| 5 | 0.695 +0.135 -0.176 | 0.364 +0.210 -0.202 | 0.891 +0.071 -0.119 | 0.701 +0.128 -0.164 | 0.665 +0.205 -0.313 | 0.690 +0.185 -0.253 | 0.463 +0.188 -0.222 | 0.774 +0.124 -0.205 |
| 7 | 0.714 +0.126 -0.171 | 0.460 +0.228 -0.239 | 0.861 +0.083 -0.145 | 0.742 +0.136 -0.181 | 0.663 +0.194 -0.276 | 0.718 +0.179 -0.267 | 0.536 +0.185 -0.233 | 0.779 +0.122 -0.207 |
| 9 | 0.727 +0.115 -0.165 | 0.536 +0.219 -0.258 | 0.838 +0.099 -0.146 | 0.765 +0.131 -0.178 | 0.667 +0.183 -0.272 | 0.740 +0.167 -0.255 | 0.587 +0.177 -0.225 | 0.782 +0.114 -0.192 |
| 11 | 0.738 +0.106 -0.159 | 0.579 +0.235 -0.269 | 0.824 +0.105 -0.162 | 0.772 +0.126 -0.189 | 0.658 +0.188 -0.271 | 0.763 +0.154 -0.248 | 0.608 +0.184 -0.224 | 0.789 +0.113 -0.202 |
| 13 | 0.740 +0.107 -0.174 | 0.617 +0.229 -0.277 | 0.805 +0.117 -0.174 | 0.779 +0.133 -0.216 | 0.657 +0.192 -0.285 | 0.768 +0.158 -0.289 | 0.628 +0.178 -0.238 | 0.782 +0.110 -0.229 |
| 15 | 0.739 +0.104 -0.154 | 0.640 +0.233 -0.291 | 0.789 +0.130 -0.204 | 0.781 +0.127 -0.197 | 0.648 +0.200 -0.248 | 0.779 +0.157 -0.266 | 0.635 +0.180 -0.232 | 0.780 +0.115 -0.205 |
| 17 | 0.742 +0.105 -0.161 | 0.655 +0.228 -0.301 | 0.785 +0.127 -0.185 | 0.784 +0.131 -0.197 | 0.643 +0.184 -0.260 | 0.788 +0.152 -0.254 | 0.640 +0.179 -0.236 | 0.782 +0.112 -0.191 |
| 19 | 0.743 +0.105 -0.157 | 0.670 +0.234 -0.298 | 0.775 +0.132 -0.189 | 0.783 +0.131 -0.197 | 0.651 +0.185 -0.260 | 0.786 +0.161 -0.270 | 0.652 +0.173 -0.233 | 0.776 +0.116 -0.200 |
| 21 | 0.747 +0.108 -0.157 | 0.680 +0.233 -0.306 | 0.774 +0.135 -0.193 | 0.788 +0.131 -0.203 | 0.632 +0.193 -0.257 | 0.804 +0.145 -0.250 | 0.646 +0.175 -0.235 | 0.785 +0.114 -0.197 |
| 23 | 0.752 +0.108 -0.157 | 0.697 +0.222 -0.304 | 0.774 +0.138 -0.185 | 0.794 +0.127 -0.194 | 0.651 +0.181 -0.257 | 0.804 +0.149 -0.280 | 0.664 +0.178 -0.235 | 0.785 +0.116 -0.201 |
| 25 | 0.752 +0.105 -0.149 | 0.710 +0.218 -0.321 | 0.763 +0.142 -0.197 | 0.793 +0.126 -0.191 | 0.648 +0.187 -0.256 | 0.810 +0.143 -0.242 | 0.670 +0.177 -0.242 | 0.782 +0.111 -0.191 |
| 27 | 0.751 +0.110 -0.162 | 0.709 +0.227 -0.319 | 0.762 +0.144 -0.222 | 0.789 +0.133 -0.216 | 0.642 +0.189 -0.264 | 0.810 +0.147 -0.286 | 0.665 +0.182 -0.243 | 0.781 +0.122 -0.218 |
| 29 | 0.751 +0.113 -0.170 | 0.719 +0.222 -0.322 | 0.759 +0.146 -0.199 | 0.791 +0.135 -0.217 | 0.643 +0.198 -0.274 | 0.816 +0.143 -0.267 | 0.671 +0.173 -0.270 | 0.782 +0.118 -0.205 |
| 31 | 0.749 +0.109 -0.163 | 0.713 +0.229 -0.289 | 0.757 +0.150 -0.202 | 0.785 +0.136 -0.208 | 0.640 +0.186 -0.235 | 0.812 +0.149 -0.265 | 0.666 +0.179 -0.215 | 0.779 +0.119 -0.205 |
| 33 | 0.752 +0.115 -0.159 | 0.725 +0.220 -0.309 | 0.754 +0.148 -0.220 | 0.788 +0.141 -0.210 | 0.639 +0.200 -0.258 | 0.820 +0.146 -0.262 | 0.671 +0.175 -0.242 | 0.780 +0.120 -0.192 |
| 35 | 0.752 +0.112 -0.165 | 0.722 +0.226 -0.316 | 0.757 +0.145 -0.224 | 0.789 +0.138 -0.212 | 0.642 +0.186 -0.238 | 0.817 +0.149 -0.254 | 0.671 +0.176 -0.225 | 0.782 +0.122 -0.209 |
| 37 | 0.756 +0.114 -0.159 | 0.737 +0.216 -0.322 | 0.755 +0.143 -0.195 | 0.794 +0.136 -0.204 | 0.643 +0.190 -0.264 | 0.826 +0.144 -0.263 | 0.678 +0.176 -0.250 | 0.785 +0.117 -0.203 |
| 39 | 0.756 +0.110 -0.173 | 0.742 +0.216 -0.313 | 0.751 +0.148 -0.225 | 0.795 +0.135 -0.222 | 0.647 +0.189 -0.244 | 0.825 +0.149 -0.260 | 0.683 +0.172 -0.236 | 0.782 +0.120 -0.210 |
| 41 | 0.757 +0.111 -0.153 | 0.746 +0.217 -0.308 | 0.750 +0.152 -0.214 | 0.795 +0.137 -0.221 | 0.642 +0.196 -0.270 | 0.829 +0.147 -0.255 | 0.681 +0.173 -0.239 | 0.782 +0.119 -0.196 |

**Supplemental Table 3**.

Performance metrics of the k-NN classifier by records (PAF as the true class) for the QT, TQ intervals with odd values of k ranging from 3 to 41. Results correspond to the means and the 95% coverage intervals of the random cross-validation runs over 1000 runs (- and + correspond to the 2.5th and 97.5th percentiles respectively), for the machine learning model. The displayed metrics are accuracy (ACC), true positive rate (TPR), true negative rate (TNR), area under the receiver operating characteristic curve (AUC), positive predictive value (PPV), negative predictive value (NPV), F_1_ score for PAF subjects (F_1_ PAF) and F_1_ score for control subjects (F_1_ CTR).

| *k* | ACC | TPR | TNR | AUC | PPV | NPV | F_1_ (PAF) | F_1_ (CTR) |
| --- | --- | --- | --- | --- | --- | --- | --- | --- |
| 3 | 0.639 +0.178 -0.211 | 0.109 +0.103 -0.078 | 0.962 +0.027 -0.055 | 0.565 +0.082 -0.075 | 0.619 +0.260 -0.356 | 0.636 +0.196 -0.238 | 0.182 +0.147 -0.125 | 0.760 +0.138 -0.199 |
| 5 | 0.663 +0.168 -0.201 | 0.193 +0.157 -0.136 | 0.943 +0.040 -0.067 | 0.641 +0.126 -0.130 | 0.652 +0.223 -0.365 | 0.657 +0.195 -0.240 | 0.292 +0.187 -0.190 | 0.769 +0.136 -0.199 |
| 7 | 0.676 +0.149 -0.166 | 0.278 +0.192 -0.170 | 0.920 +0.052 -0.081 | 0.689 +0.128 -0.148 | 0.669 +0.215 -0.326 | 0.668 +0.190 -0.242 | 0.386 +0.207 -0.213 | 0.769 +0.131 -0.194 |
| 9 | 0.711 +0.128 -0.181 | 0.373 +0.236 -0.219 | 0.909 +0.054 -0.089 | 0.734 +0.134 -0.161 | 0.698 +0.189 -0.310 | 0.702 +0.165 -0.243 | 0.478 +0.216 -0.249 | 0.789 +0.116 -0.188 |
| 11 | 0.728 +0.118 -0.158 | 0.434 +0.231 -0.234 | 0.896 +0.062 -0.096 | 0.763 +0.128 -0.172 | 0.699 +0.193 -0.301 | 0.723 +0.162 -0.248 | 0.528 +0.205 -0.241 | 0.796 +0.110 -0.182 |
| 13 | 0.738 +0.115 -0.174 | 0.500 +0.248 -0.259 | 0.876 +0.074 -0.117 | 0.782 +0.124 -0.174 | 0.701 +0.181 -0.276 | 0.738 +0.168 -0.255 | 0.575 +0.197 -0.241 | 0.797 +0.110 -0.207 |
| 15 | 0.753 +0.111 -0.157 | 0.553 +0.249 -0.276 | 0.867 +0.080 -0.111 | 0.794 +0.120 -0.178 | 0.704 +0.172 -0.256 | 0.759 +0.164 -0.254 | 0.611 +0.186 -0.234 | 0.805 +0.109 -0.191 |
| 17 | 0.766 +0.100 -0.162 | 0.603 +0.240 -0.301 | 0.857 +0.079 -0.114 | 0.808 +0.114 -0.187 | 0.708 +0.163 -0.263 | 0.780 +0.151 -0.251 | 0.642 +0.177 -0.249 | 0.813 +0.097 -0.177 |
| 19 | 0.770 +0.101 -0.156 | 0.636 +0.232 -0.314 | 0.844 +0.088 -0.130 | 0.808 +0.117 -0.192 | 0.707 +0.176 -0.257 | 0.786 +0.153 -0.256 | 0.661 +0.174 -0.246 | 0.810 +0.102 -0.183 |
| 21 | 0.775 +0.106 -0.150 | 0.652 +0.236 -0.316 | 0.840 +0.092 -0.144 | 0.811 +0.124 -0.184 | 0.709 +0.154 -0.259 | 0.795 +0.152 -0.267 | 0.671 +0.175 -0.246 | 0.813 +0.100 -0.189 |
| 23 | 0.777 +0.106 -0.162 | 0.670 +0.235 -0.326 | 0.836 +0.094 -0.134 | 0.815 +0.124 -0.205 | 0.708 +0.165 -0.273 | 0.801 +0.155 -0.246 | 0.679 +0.176 -0.250 | 0.814 +0.099 -0.178 |
| 25 | 0.782 +0.098 -0.150 | 0.696 +0.226 -0.320 | 0.827 +0.095 -0.152 | 0.821 +0.116 -0.180 | 0.709 +0.164 -0.246 | 0.811 +0.147 -0.257 | 0.694 +0.167 -0.240 | 0.815 +0.097 -0.184 |
| 27 | 0.788 +0.098 -0.145 | 0.704 +0.227 -0.331 | 0.829 +0.100 -0.135 | 0.824 +0.114 -0.192 | 0.707 +0.175 -0.257 | 0.821 +0.140 -0.253 | 0.697 +0.175 -0.255 | 0.821 +0.096 -0.166 |
| 29 | 0.789 +0.099 -0.136 | 0.717 +0.218 -0.316 | 0.825 +0.109 -0.138 | 0.827 +0.112 -0.186 | 0.705 +0.167 -0.258 | 0.828 +0.137 -0.234 | 0.702 +0.164 -0.243 | 0.823 +0.096 -0.151 |
| 31 | 0.787 +0.100 -0.145 | 0.717 +0.223 -0.323 | 0.822 +0.103 -0.149 | 0.824 +0.115 -0.185 | 0.708 +0.160 -0.244 | 0.823 +0.143 -0.256 | 0.704 +0.159 -0.237 | 0.819 +0.098 -0.179 |
| 33 | 0.787 +0.098 -0.156 | 0.722 +0.226 -0.334 | 0.818 +0.111 -0.149 | 0.825 +0.116 -0.191 | 0.707 +0.167 -0.262 | 0.825 +0.143 -0.242 | 0.705 +0.175 -0.235 | 0.817 +0.097 -0.173 |
| 35 | 0.788 +0.102 -0.171 | 0.731 +0.218 -0.345 | 0.816 +0.110 -0.140 | 0.829 +0.115 -0.209 | 0.705 +0.160 -0.225 | 0.829 +0.141 -0.263 | 0.709 +0.165 -0.232 | 0.818 +0.099 -0.182 |
| 37 | 0.787 +0.099 -0.164 | 0.734 +0.214 -0.343 | 0.811 +0.108 -0.156 | 0.828 +0.114 -0.196 | 0.702 +0.163 -0.243 | 0.829 +0.139 -0.268 | 0.709 +0.164 -0.248 | 0.816 +0.101 -0.183 |
| 39 | 0.788 +0.098 -0.143 | 0.737 +0.208 -0.319 | 0.811 +0.114 -0.165 | 0.830 +0.113 -0.191 | 0.704 +0.161 -0.232 | 0.831 +0.142 -0.258 | 0.712 +0.159 -0.232 | 0.817 +0.102 -0.184 |
| 41 | 0.790 +0.096 -0.136 | 0.743 +0.211 -0.300 | 0.809 +0.108 -0.146 | 0.832 +0.113 -0.193 | 0.699 +0.160 -0.234 | 0.836 +0.138 -0.241 | 0.712 +0.159 -0.228 | 0.819 +0.096 -0.171 |

**Supplemental Table 4**.

Performance metrics of the k-NN classifier by records (PAF as the true class) for the RR, QT, TQ intervals with odd values of k ranging from 3 to 41. Results correspond to the means and the 95% coverage intervals of the random cross-validation runs over 1000 runs (- and + correspond to the 2.5th and 97.5th percentiles respectively), for the machine learning model. The displayed metrics are accuracy (ACC), true positive rate (TPR), true negative rate (TNR), area under the receiver operating characteristic curve (AUC), positive predictive value (PPV), negative predictive value (NPV), F_1_ score for PAF subjects (F_1_ PAF) and F_1_ score for control subjects (F_1_ CTR).

| *k* | ACC | TPR | TNR | AUC | PPV | NPV | F_1_ (PAF) | F_1_ (CTR) |
| --- | --- | --- | --- | --- | --- | --- | --- | --- |
| 3 | 0.740 +0.096 -0.166 | 0.553 +0.195 -0.283 | 0.853 +0.082 -0.099 | 0.750 +0.112 -0.151 | 0.687 +0.181 -0.281 | 0.750 +0.165 -0.259 | 0.603 +0.165 -0.224 | 0.794 +0.102 -0.186 |
| 5 | 0.755 +0.090 -0.150 | 0.615 +0.213 -0.305 | 0.835 +0.098 -0.115 | 0.777 +0.118 -0.168 | 0.680 +0.179 -0.260 | 0.781 +0.154 -0.248 | 0.635 +0.169 -0.231 | 0.803 +0.094 -0.166 |
| 7 | 0.763 +0.091 -0.133 | 0.659 +0.213 -0.306 | 0.822 +0.101 -0.125 | 0.792 +0.116 -0.164 | 0.687 +0.178 -0.244 | 0.796 +0.148 -0.248 | 0.663 +0.162 -0.224 | 0.804 +0.092 -0.163 |
| 9 | 0.769 +0.085 -0.131 | 0.679 +0.216 -0.302 | 0.816 +0.111 -0.137 | 0.798 +0.117 -0.165 | 0.687 +0.173 -0.245 | 0.803 +0.147 -0.253 | 0.674 +0.165 -0.231 | 0.805 +0.097 -0.173 |
| 11 | 0.765 +0.094 -0.143 | 0.686 +0.226 -0.332 | 0.810 +0.108 -0.133 | 0.797 +0.125 -0.184 | 0.683 +0.174 -0.266 | 0.805 +0.153 -0.249 | 0.673 +0.174 -0.237 | 0.802 +0.097 -0.162 |
| 13 | 0.769 +0.101 -0.149 | 0.702 +0.217 -0.336 | 0.804 +0.112 -0.147 | 0.802 +0.124 -0.184 | 0.685 +0.168 -0.264 | 0.810 +0.154 -0.272 | 0.684 +0.168 -0.241 | 0.802 +0.104 -0.197 |
| 15 | 0.776 +0.092 -0.137 | 0.721 +0.211 -0.342 | 0.803 +0.118 -0.145 | 0.811 +0.117 -0.182 | 0.683 +0.170 -0.253 | 0.825 +0.141 -0.247 | 0.691 +0.163 -0.236 | 0.810 +0.093 -0.159 |
| 17 | 0.777 +0.092 -0.144 | 0.729 +0.214 -0.321 | 0.800 +0.127 -0.157 | 0.814 +0.117 -0.182 | 0.683 +0.176 -0.270 | 0.827 +0.144 -0.273 | 0.695 +0.173 -0.240 | 0.808 +0.098 -0.172 |
| 19 | 0.774 +0.092 -0.157 | 0.725 +0.222 -0.344 | 0.797 +0.117 -0.151 | 0.808 +0.123 -0.222 | 0.679 +0.171 -0.253 | 0.825 +0.146 -0.288 | 0.691 +0.164 -0.248 | 0.806 +0.098 -0.192 |
| 21 | 0.780 +0.087 -0.138 | 0.736 +0.208 -0.311 | 0.800 +0.111 -0.150 | 0.815 +0.115 -0.185 | 0.685 +0.173 -0.246 | 0.832 +0.137 -0.263 | 0.700 +0.163 -0.233 | 0.811 +0.093 -0.157 |
| 23 | 0.778 +0.093 -0.148 | 0.736 +0.213 -0.333 | 0.797 +0.120 -0.138 | 0.813 +0.121 -0.197 | 0.690 +0.163 -0.236 | 0.827 +0.145 -0.267 | 0.703 +0.158 -0.228 | 0.807 +0.097 -0.180 |
| 25 | 0.781 +0.096 -0.144 | 0.741 +0.216 -0.302 | 0.798 +0.122 -0.155 | 0.817 +0.124 -0.195 | 0.688 +0.157 -0.222 | 0.834 +0.142 -0.241 | 0.704 +0.156 -0.218 | 0.811 +0.095 -0.166 |
| 27 | 0.779 +0.093 -0.146 | 0.740 +0.215 -0.345 | 0.794 +0.126 -0.153 | 0.814 +0.126 -0.194 | 0.685 +0.168 -0.239 | 0.831 +0.143 -0.258 | 0.702 +0.159 -0.234 | 0.808 +0.097 -0.176 |
| 29 | 0.782 +0.095 -0.148 | 0.752 +0.205 -0.313 | 0.794 +0.121 -0.159 | 0.820 +0.120 -0.199 | 0.687 +0.169 -0.230 | 0.840 +0.134 -0.259 | 0.709 +0.169 -0.217 | 0.812 +0.098 -0.164 |
| 31 | 0.784 +0.093 -0.143 | 0.756 +0.206 -0.308 | 0.791 +0.120 -0.148 | 0.822 +0.117 -0.200 | 0.683 +0.167 -0.246 | 0.841 +0.136 -0.259 | 0.708 +0.166 -0.243 | 0.811 +0.097 -0.167 |
| 33 | 0.785 +0.099 -0.138 | 0.750 +0.209 -0.300 | 0.799 +0.117 -0.148 | 0.822 +0.123 -0.191 | 0.688 +0.167 -0.228 | 0.838 +0.139 -0.269 | 0.709 +0.160 -0.228 | 0.813 +0.099 -0.178 |
| 35 | 0.781 +0.097 -0.146 | 0.750 +0.209 -0.339 | 0.793 +0.124 -0.157 | 0.820 +0.125 -0.196 | 0.683 +0.164 -0.244 | 0.839 +0.139 -0.257 | 0.705 +0.166 -0.229 | 0.810 +0.097 -0.170 |
| 37 | 0.786 +0.092 -0.144 | 0.765 +0.196 -0.306 | 0.791 +0.115 -0.148 | 0.827 +0.114 -0.192 | 0.687 +0.164 -0.252 | 0.844 +0.134 -0.261 | 0.714 +0.157 -0.221 | 0.812 +0.096 -0.171 |
| 39 | 0.783 +0.097 -0.146 | 0.758 +0.207 -0.337 | 0.791 +0.120 -0.153 | 0.824 +0.121 -0.198 | 0.682 +0.164 -0.236 | 0.842 +0.138 -0.268 | 0.708 +0.163 -0.236 | 0.811 +0.098 -0.171 |
| 41 | 0.781 +0.093 -0.144 | 0.758 +0.205 -0.306 | 0.788 +0.127 -0.166 | 0.823 +0.124 -0.192 | 0.684 +0.156 -0.236 | 0.840 +0.140 -0.260 | 0.709 +0.164 -0.219 | 0.808 +0.098 -0.179 |

**Supplemental Table 5**.

Performance metrics of the k-NN classifier by majority votes (PAF as the true class) for the RR, QT intervals with odd values of k ranging from 3 to 41. Results correspond to the means and the 95% coverage intervals of the random cross-validation runs over 1000 runs (- and + correspond to the 2.5th and 97.5th percentiles respectively), for the machine learning model. The displayed metrics are accuracy (ACC), true positive rate (TPR), true negative rate (TNR), area under the receiver operating characteristic curve (AUC), positive predictive value (PPV), negative predictive value (NPV), F_1_ score for PAF subjects (F_1_ PAF) and F_1_ score for control subjects (F_1_ CTR).

| *k* | ACC | TPR | TNR | AUC | PPV | NPV | F_1_ (PAF) | F_1_ (CTR) |
| --- | --- | --- | --- | --- | --- | --- | --- | --- |
| 3 | 0.700 +0.000 -0.000 | 0.000 +0.000 -0.000 | 1.000 +0.000 -0.000 | 0.708 +0.292 -0.327 | 0.000 +0.000 -0.000 | 0.700 +0.000 -0.000 | 0.000 +0.000 -0.000 | 0.824 +0.000 -0.000 |
| 5 | 0.700 +0.000 -0.000 | 0.004 +0.004 -0.004 | 0.998 +0.002 -0.002 | 0.759 +0.241 -0.330 | 0.011 +0.011 -0.011 | 0.700 +0.000 -0.000 | 0.005 +0.005 -0.005 | 0.823 +0.001 -0.001 |
| 7 | 0.715 +0.085 -0.115 | 0.108 +0.226 -0.108 | 0.976 +0.024 -0.119 | 0.818 +0.182 -0.341 | 0.288 +0.712 -0.288 | 0.720 +0.058 -0.053 | 0.154 +0.346 -0.154 | 0.828 +0.047 -0.078 |
| 9 | 0.742 +0.158 -0.142 | 0.249 +0.418 -0.249 | 0.954 +0.046 -0.240 | 0.836 +0.164 -0.313 | 0.529 +0.471 -0.529 | 0.751 +0.124 -0.084 | 0.326 +0.474 -0.326 | 0.838 +0.095 -0.124 |
| 11 | 0.755 +0.145 -0.155 | 0.317 +0.350 -0.317 | 0.943 +0.057 -0.229 | 0.867 +0.133 -0.248 | 0.604 +0.396 -0.604 | 0.767 +0.108 -0.101 | 0.399 +0.401 -0.399 | 0.844 +0.089 -0.130 |
| 13 | 0.769 +0.231 -0.269 | 0.425 +0.575 -0.425 | 0.917 +0.083 -0.203 | 0.862 +0.138 -0.290 | 0.655 +0.345 -0.655 | 0.794 +0.206 -0.169 | 0.492 +0.508 -0.492 | 0.848 +0.152 -0.181 |
| 15 | 0.810 +0.190 -0.210 | 0.598 +0.402 -0.598 | 0.901 +0.099 -0.186 | 0.876 +0.124 -0.257 | 0.728 +0.272 -0.728 | 0.847 +0.153 -0.180 | 0.631 +0.369 -0.631 | 0.868 +0.132 -0.202 |
| 17 | 0.821 +0.179 -0.221 | 0.718 +0.282 -0.718 | 0.865 +0.135 -0.294 | 0.888 +0.112 -0.269 | 0.712 +0.288 -0.712 | 0.888 +0.112 -0.204 | 0.690 +0.310 -0.690 | 0.870 +0.130 -0.203 |
| 19 | 0.826 +0.174 -0.226 | 0.754 +0.246 -0.421 | 0.857 +0.143 -0.286 | 0.889 +0.111 -0.270 | 0.722 +0.278 -0.389 | 0.900 +0.100 -0.186 | 0.714 +0.286 -0.380 | 0.871 +0.129 -0.157 |
| 21 | 0.825 +0.175 -0.225 | 0.753 +0.247 -0.420 | 0.855 +0.145 -0.284 | 0.892 +0.108 -0.272 | 0.722 +0.278 -0.388 | 0.898 +0.102 -0.184 | 0.715 +0.285 -0.382 | 0.870 +0.130 -0.203 |
| 23 | 0.829 +0.171 -0.229 | 0.768 +0.232 -0.434 | 0.855 +0.145 -0.284 | 0.897 +0.103 -0.254 | 0.726 +0.274 -0.393 | 0.903 +0.097 -0.189 | 0.725 +0.275 -0.391 | 0.872 +0.128 -0.205 |
| 25 | 0.824 +0.176 -0.224 | 0.779 +0.221 -0.446 | 0.844 +0.156 -0.272 | 0.897 +0.103 -0.278 | 0.709 +0.291 -0.442 | 0.907 +0.093 -0.207 | 0.722 +0.278 -0.412 | 0.868 +0.132 -0.201 |
| 27 | 0.825 +0.175 -0.225 | 0.783 +0.217 -0.450 | 0.842 +0.158 -0.271 | 0.900 +0.100 -0.233 | 0.711 +0.289 -0.377 | 0.909 +0.091 -0.194 | 0.724 +0.276 -0.390 | 0.868 +0.132 -0.201 |
| 29 | 0.820 +0.180 -0.220 | 0.797 +0.203 -0.463 | 0.831 +0.169 -0.259 | 0.903 +0.097 -0.236 | 0.702 +0.298 -0.368 | 0.914 +0.086 -0.200 | 0.724 +0.276 -0.390 | 0.862 +0.138 -0.196 |
| 31 | 0.831 +0.169 -0.231 | 0.808 +0.192 -0.475 | 0.840 +0.160 -0.269 | 0.903 +0.097 -0.260 | 0.719 +0.281 -0.385 | 0.919 +0.081 -0.204 | 0.740 +0.260 -0.407 | 0.871 +0.129 -0.204 |
| 33 | 0.820 +0.180 -0.220 | 0.801 +0.199 -0.467 | 0.828 +0.172 -0.257 | 0.901 +0.099 -0.235 | 0.698 +0.302 -0.365 | 0.914 +0.086 -0.200 | 0.726 +0.274 -0.392 | 0.862 +0.138 -0.195 |
| 35 | 0.827 +0.173 -0.227 | 0.813 +0.187 -0.480 | 0.832 +0.168 -0.261 | 0.903 +0.097 -0.237 | 0.704 +0.296 -0.371 | 0.920 +0.080 -0.205 | 0.736 +0.264 -0.403 | 0.867 +0.133 -0.200 |
| 37 | 0.820 +0.180 -0.220 | 0.823 +0.177 -0.489 | 0.819 +0.181 -0.247 | 0.900 +0.100 -0.281 | 0.696 +0.304 -0.363 | 0.922 +0.078 -0.207 | 0.734 +0.266 -0.401 | 0.859 +0.141 -0.244 |
| 39 | 0.822 +0.178 -0.222 | 0.832 +0.168 -0.499 | 0.818 +0.182 -0.247 | 0.903 +0.097 -0.284 | 0.696 +0.304 -0.363 | 0.927 +0.073 -0.213 | 0.738 +0.262 -0.404 | 0.861 +0.139 -0.194 |
| 41 | 0.820 +0.180 -0.220 | 0.827 +0.173 -0.494 | 0.817 +0.183 -0.246 | 0.902 +0.098 -0.283 | 0.696 +0.304 -0.363 | 0.925 +0.075 -0.211 | 0.735 +0.265 -0.401 | 0.860 +0.140 -0.193 |

**Supplemental Table 6**.

Performance metrics of the k-NN classifier by majority votes (PAF as the true class) for the RR, TQ intervals with odd values of k ranging from 3 to 41. Results correspond to the means and the 95% coverage intervals of the random cross-validation runs over 1000 runs (- and + correspond to the 2.5th and 97.5th percentiles respectively), for the machine learning model. The displayed metrics are accuracy (ACC), true positive rate (TPR), true negative rate (TNR), area under the receiver operating characteristic curve (AUC), positive predictive value (PPV), negative predictive value (NPV), F_1_ score for PAF subjects (F_1_ PAF) and F_1_ score for control subjects (F_1_ CTR).

| *k* | ACC | TPR | TNR | AUC | PPV | NPV | F_1_ (PAF) | F_1_ (CTR) |
| --- | --- | --- | --- | --- | --- | --- | --- | --- |
| 3 | 0.692 +0.108 -0.092 | 0.013 +0.320 -0.013 | 0.982 +0.018 -0.125 | 0.755 +0.245 -0.327 | 0.038 +0.962 -0.038 | 0.699 +0.079 -0.032 | 0.020 +0.480 -0.020 | 0.817 +0.058 -0.067 |
| 5 | 0.741 +0.159 -0.141 | 0.250 +0.417 -0.250 | 0.952 +0.048 -0.238 | 0.788 +0.212 -0.312 | 0.511 +0.489 -0.511 | 0.751 +0.124 -0.084 | 0.323 +0.477 -0.323 | 0.837 +0.096 -0.123 |
| 7 | 0.746 +0.154 -0.246 | 0.465 +0.535 -0.465 | 0.867 +0.133 -0.296 | 0.786 +0.214 -0.310 | 0.614 +0.386 -0.614 | 0.796 +0.204 -0.129 | 0.502 +0.355 -0.502 | 0.824 +0.109 -0.209 |
| 9 | 0.733 +0.267 -0.233 | 0.606 +0.394 -0.606 | 0.788 +0.212 -0.359 | 0.808 +0.192 -0.285 | 0.571 +0.429 -0.571 | 0.833 +0.167 -0.208 | 0.561 +0.439 -0.561 | 0.800 +0.200 -0.255 |
| 11 | 0.742 +0.258 -0.242 | 0.729 +0.271 -0.396 | 0.748 +0.252 -0.319 | 0.818 +0.182 -0.294 | 0.585 +0.415 -0.385 | 0.874 +0.126 -0.207 | 0.628 +0.372 -0.378 | 0.795 +0.205 -0.250 |
| 13 | 0.723 +0.277 -0.273 | 0.753 +0.247 -0.419 | 0.711 +0.289 -0.282 | 0.815 +0.185 -0.292 | 0.556 +0.444 -0.356 | 0.879 +0.121 -0.212 | 0.620 +0.380 -0.370 | 0.774 +0.226 -0.274 |
| 15 | 0.724 +0.226 -0.274 | 0.767 +0.233 -0.434 | 0.705 +0.295 -0.420 | 0.818 +0.182 -0.295 | 0.557 +0.443 -0.307 | 0.886 +0.114 -0.219 | 0.626 +0.303 -0.340 | 0.772 +0.195 -0.327 |
| 17 | 0.722 +0.228 -0.322 | 0.776 +0.224 -0.443 | 0.699 +0.301 -0.271 | 0.817 +0.183 -0.293 | 0.550 +0.450 -0.350 | 0.889 +0.111 -0.264 | 0.626 +0.302 -0.376 | 0.771 +0.196 -0.309 |
| 19 | 0.716 +0.284 -0.316 | 0.780 +0.220 -0.447 | 0.689 +0.311 -0.403 | 0.810 +0.190 -0.286 | 0.546 +0.454 -0.296 | 0.888 +0.112 -0.222 | 0.626 +0.374 -0.340 | 0.763 +0.237 -0.318 |
| 21 | 0.725 +0.275 -0.225 | 0.804 +0.196 -0.470 | 0.692 +0.308 -0.263 | 0.818 +0.182 -0.294 | 0.554 +0.446 -0.304 | 0.903 +0.097 -0.236 | 0.638 +0.362 -0.352 | 0.770 +0.230 -0.270 |
| 23 | 0.733 +0.267 -0.233 | 0.823 +0.177 -0.490 | 0.695 +0.305 -0.266 | 0.823 +0.177 -0.299 | 0.564 +0.436 -0.278 | 0.908 +0.092 -0.242 | 0.655 +0.345 -0.321 | 0.776 +0.224 -0.231 |
| 25 | 0.731 +0.269 -0.231 | 0.836 +0.164 -0.503 | 0.686 +0.314 -0.258 | 0.816 +0.184 -0.292 | 0.556 +0.444 -0.270 | 0.918 +0.082 -0.251 | 0.653 +0.347 -0.320 | 0.772 +0.228 -0.272 |
| 27 | 0.727 +0.273 -0.227 | 0.840 +0.160 -0.507 | 0.679 +0.321 -0.393 | 0.815 +0.185 -0.292 | 0.556 +0.444 -0.306 | 0.917 +0.083 -0.250 | 0.653 +0.347 -0.344 | 0.767 +0.233 -0.322 |
| 29 | 0.731 +0.269 -0.231 | 0.843 +0.157 -0.510 | 0.682 +0.318 -0.397 | 0.813 +0.187 -0.289 | 0.558 +0.442 -0.290 | 0.919 +0.081 -0.252 | 0.657 +0.343 -0.324 | 0.770 +0.230 -0.326 |
| 31 | 0.728 +0.272 -0.328 | 0.848 +0.152 -0.514 | 0.676 +0.324 -0.390 | 0.806 +0.194 -0.330 | 0.560 +0.440 -0.274 | 0.918 +0.082 -0.252 | 0.659 +0.341 -0.259 | 0.765 +0.235 -0.320 |
| 33 | 0.736 +0.264 -0.236 | 0.856 +0.144 -0.189 | 0.685 +0.315 -0.399 | 0.812 +0.188 -0.288 | 0.566 +0.434 -0.233 | 0.924 +0.076 -0.210 | 0.668 +0.332 -0.268 | 0.773 +0.227 -0.329 |
| 35 | 0.734 +0.266 -0.234 | 0.849 +0.151 -0.516 | 0.684 +0.316 -0.399 | 0.809 +0.191 -0.285 | 0.564 +0.436 -0.230 | 0.922 +0.078 -0.208 | 0.663 +0.337 -0.330 | 0.771 +0.229 -0.327 |
| 37 | 0.737 +0.263 -0.237 | 0.865 +0.135 -0.531 | 0.682 +0.318 -0.396 | 0.815 +0.185 -0.339 | 0.566 +0.434 -0.233 | 0.929 +0.071 -0.215 | 0.669 +0.331 -0.269 | 0.774 +0.226 -0.329 |
| 39 | 0.739 +0.261 -0.239 | 0.862 +0.138 -0.529 | 0.686 +0.314 -0.257 | 0.813 +0.187 -0.289 | 0.569 +0.431 -0.236 | 0.927 +0.073 -0.212 | 0.671 +0.329 -0.271 | 0.776 +0.224 -0.295 |
| 41 | 0.731 +0.269 -0.231 | 0.865 +0.135 -0.532 | 0.673 +0.327 -0.388 | 0.810 +0.190 -0.286 | 0.558 +0.442 -0.225 | 0.930 +0.070 -0.216 | 0.664 +0.336 -0.298 | 0.767 +0.233 -0.322 |

**Supplemental Table 7**.

Performance metrics of the k-NN classifier by majority votes (PAF as the true class) for the QT, TQ intervals with odd values of k ranging from 3 to 41. Results correspond to the means and the 95% coverage intervals of the random cross-validation runs over 1000 runs (- and + correspond to the 2.5th and 97.5th percentiles respectively), for the machine learning model. The displayed metrics are accuracy (ACC), true positive rate (TPR), true negative rate (TNR), area under the receiver operating characteristic curve (AUC), positive predictive value (PPV), negative predictive value (NPV), F_1_ score for PAF subjects (F_1_ PAF) and F_1_ score for control subjects (F_1_ CTR).

| *k* | ACC | TPR | TNR | AUC | PPV | NPV | F_1_ (PAF) | F_1_ (CTR) |
| --- | --- | --- | --- | --- | --- | --- | --- | --- |
| 3 | 0.700 +0.000 -0.000 | 0.000 +0.000 -0.000 | 1.000 +0.000 -0.000 | 0.685 +0.315 -0.352 | 0.000 +0.000 -0.000 | 0.700 +0.000 -0.000 | 0.000 +0.000 -0.000 | 0.823 +0.000 -0.000 |
| 5 | 0.700 +0.000 -0.000 | 0.005 +0.005 -0.005 | 0.997 +0.003 -0.003 | 0.735 +0.265 -0.354 | 0.016 +0.016 -0.016 | 0.701 +0.001 -0.001 | 0.008 +0.008 -0.008 | 0.823 +0.001 -0.001 |
| 7 | 0.701 +0.099 -0.101 | 0.088 +0.245 -0.088 | 0.963 +0.037 -0.106 | 0.766 +0.234 -0.290 | 0.224 +0.776 -0.224 | 0.712 +0.065 -0.046 | 0.124 +0.376 -0.124 | 0.818 +0.057 -0.068 |
| 9 | 0.741 +0.159 -0.141 | 0.258 +0.408 -0.258 | 0.948 +0.052 -0.234 | 0.819 +0.181 -0.343 | 0.537 +0.463 -0.537 | 0.752 +0.123 -0.085 | 0.337 +0.463 -0.337 | 0.837 +0.096 -0.123 |
| 11 | 0.755 +0.145 -0.155 | 0.334 +0.666 -0.334 | 0.935 +0.065 -0.221 | 0.843 +0.157 -0.272 | 0.611 +0.389 -0.611 | 0.771 +0.229 -0.104 | 0.413 +0.415 -0.413 | 0.843 +0.091 -0.128 |
| 13 | 0.774 +0.226 -0.174 | 0.458 +0.542 -0.458 | 0.909 +0.091 -0.195 | 0.856 +0.144 -0.284 | 0.669 +0.331 -0.669 | 0.804 +0.196 -0.137 | 0.517 +0.483 -0.517 | 0.849 +0.151 -0.182 |
| 15 | 0.793 +0.207 -0.193 | 0.611 +0.389 -0.611 | 0.872 +0.128 -0.300 | 0.863 +0.137 -0.244 | 0.684 +0.316 -0.684 | 0.849 +0.151 -0.182 | 0.619 +0.381 -0.619 | 0.854 +0.146 -0.187 |
| 17 | 0.815 +0.185 -0.215 | 0.735 +0.265 -0.735 | 0.850 +0.150 -0.278 | 0.881 +0.119 -0.261 | 0.698 +0.302 -0.698 | 0.892 +0.108 -0.226 | 0.692 +0.308 -0.692 | 0.863 +0.137 -0.196 |
| 19 | 0.819 +0.181 -0.219 | 0.770 +0.230 -0.437 | 0.839 +0.161 -0.268 | 0.886 +0.114 -0.267 | 0.702 +0.298 -0.369 | 0.904 +0.096 -0.190 | 0.712 +0.288 -0.379 | 0.863 +0.137 -0.197 |
| 21 | 0.820 +0.180 -0.220 | 0.781 +0.219 -0.448 | 0.837 +0.163 -0.265 | 0.889 +0.111 -0.270 | 0.709 +0.291 -0.375 | 0.908 +0.092 -0.194 | 0.720 +0.280 -0.386 | 0.863 +0.137 -0.196 |
| 23 | 0.814 +0.186 -0.214 | 0.765 +0.235 -0.432 | 0.835 +0.165 -0.263 | 0.887 +0.113 -0.268 | 0.695 +0.305 -0.362 | 0.900 +0.100 -0.186 | 0.708 +0.292 -0.374 | 0.859 +0.141 -0.193 |
| 25 | 0.816 +0.184 -0.216 | 0.790 +0.210 -0.457 | 0.828 +0.172 -0.256 | 0.895 +0.105 -0.228 | 0.698 +0.302 -0.365 | 0.909 +0.091 -0.195 | 0.720 +0.280 -0.386 | 0.859 +0.141 -0.192 |
| 27 | 0.812 +0.188 -0.212 | 0.785 +0.215 -0.452 | 0.824 +0.176 -0.253 | 0.901 +0.099 -0.235 | 0.693 +0.307 -0.360 | 0.909 +0.091 -0.195 | 0.713 +0.287 -0.379 | 0.856 +0.144 -0.189 |
| 29 | 0.815 +0.185 -0.215 | 0.798 +0.202 -0.465 | 0.823 +0.177 -0.251 | 0.905 +0.095 -0.238 | 0.697 +0.303 -0.363 | 0.912 +0.088 -0.198 | 0.723 +0.277 -0.356 | 0.857 +0.143 -0.216 |
| 31 | 0.814 +0.186 -0.214 | 0.796 +0.204 -0.462 | 0.822 +0.178 -0.250 | 0.899 +0.101 -0.280 | 0.693 +0.307 -0.360 | 0.911 +0.089 -0.197 | 0.719 +0.281 -0.386 | 0.856 +0.144 -0.190 |
| 33 | 0.817 +0.183 -0.217 | 0.808 +0.192 -0.475 | 0.821 +0.179 -0.250 | 0.901 +0.099 -0.234 | 0.702 +0.298 -0.348 | 0.917 +0.083 -0.167 | 0.727 +0.273 -0.327 | 0.858 +0.142 -0.191 |
| 35 | 0.818 +0.182 -0.218 | 0.816 +0.184 -0.483 | 0.819 +0.181 -0.247 | 0.899 +0.101 -0.280 | 0.695 +0.305 -0.361 | 0.920 +0.080 -0.206 | 0.729 +0.271 -0.396 | 0.858 +0.142 -0.192 |
| 37 | 0.818 +0.182 -0.218 | 0.825 +0.175 -0.491 | 0.815 +0.185 -0.243 | 0.897 +0.103 -0.278 | 0.687 +0.313 -0.354 | 0.923 +0.077 -0.208 | 0.732 +0.268 -0.399 | 0.857 +0.143 -0.191 |
| 39 | 0.824 +0.176 -0.224 | 0.830 +0.170 -0.497 | 0.822 +0.178 -0.251 | 0.897 +0.103 -0.230 | 0.702 +0.298 -0.336 | 0.926 +0.074 -0.176 | 0.741 +0.259 -0.341 | 0.863 +0.137 -0.196 |
| 41 | 0.822 +0.178 -0.222 | 0.843 +0.157 -0.509 | 0.814 +0.186 -0.242 | 0.901 +0.099 -0.234 | 0.696 +0.304 -0.362 | 0.929 +0.071 -0.179 | 0.744 +0.256 -0.344 | 0.860 +0.140 -0.219 |

**Supplemental Table 8**.

Performance metrics of the k-NN classifier by majority votes (PAF as the true class) for the RR, QT, TQ intervals with odd values of k ranging from 3 to 41. Results correspond to the means and the 95% coverage intervals of the random cross-validation runs over 1000 runs (- and + correspond to the 2.5th and 97.5th percentiles respectively), for the machine learning model. The displayed metrics are accuracy (ACC), true positive rate (TPR), true negative rate (TNR), area under the receiver operating characteristic curve (AUC), positive predictive value (PPV), negative predictive value (NPV), F_1_ score for PAF subjects (F_1_ PAF) and F_1_ score for control subjects (F_1_ CTR).

| *k* | ACC | TPR | TNR | AUC | PPV | NPV | F_1_ (PAF) | F_1_ (CTR) |
| --- | --- | --- | --- | --- | --- | --- | --- | --- |
| 3 | 0.834 +0.166 -0.234 | 0.727 +0.273 -0.394 | 0.880 +0.120 -0.308 | 0.898 +0.102 -0.278 | 0.748 +0.252 -0.498 | 0.891 +0.109 -0.191 | 0.713 +0.287 -0.428 | 0.880 +0.120 -0.166 |
| 5 | 0.819 +0.181 -0.219 | 0.751 +0.249 -0.417 | 0.849 +0.151 -0.277 | 0.889 +0.111 -0.270 | 0.711 +0.289 -0.377 | 0.896 +0.104 -0.182 | 0.708 +0.292 -0.375 | 0.866 +0.134 -0.199 |
| 7 | 0.829 +0.171 -0.229 | 0.772 +0.228 -0.439 | 0.853 +0.147 -0.282 | 0.904 +0.096 -0.237 | 0.727 +0.273 -0.393 | 0.905 +0.095 -0.191 | 0.727 +0.273 -0.393 | 0.872 +0.128 -0.205 |
| 9 | 0.820 +0.180 -0.220 | 0.782 +0.218 -0.449 | 0.836 +0.164 -0.264 | 0.897 +0.103 -0.231 | 0.712 +0.288 -0.378 | 0.908 +0.092 -0.158 | 0.722 +0.278 -0.322 | 0.862 +0.138 -0.195 |
| 11 | 0.811 +0.189 -0.211 | 0.769 +0.231 -0.435 | 0.830 +0.170 -0.258 | 0.894 +0.106 -0.228 | 0.692 +0.308 -0.359 | 0.902 +0.098 -0.188 | 0.706 +0.294 -0.372 | 0.857 +0.143 -0.190 |
| 13 | 0.819 +0.181 -0.219 | 0.781 +0.219 -0.448 | 0.835 +0.165 -0.264 | 0.897 +0.103 -0.278 | 0.708 +0.292 -0.375 | 0.907 +0.093 -0.193 | 0.720 +0.280 -0.386 | 0.862 +0.138 -0.195 |
| 15 | 0.817 +0.183 -0.217 | 0.794 +0.206 -0.461 | 0.827 +0.173 -0.255 | 0.904 +0.096 -0.237 | 0.702 +0.298 -0.369 | 0.912 +0.088 -0.198 | 0.721 +0.279 -0.388 | 0.859 +0.141 -0.192 |
| 17 | 0.816 +0.184 -0.216 | 0.803 +0.197 -0.469 | 0.821 +0.179 -0.393 | 0.900 +0.100 -0.281 | 0.699 +0.301 -0.365 | 0.914 +0.086 -0.200 | 0.725 +0.275 -0.392 | 0.857 +0.143 -0.257 |
| 19 | 0.806 +0.194 -0.206 | 0.795 +0.205 -0.461 | 0.811 +0.189 -0.382 | 0.891 +0.109 -0.271 | 0.681 +0.319 -0.348 | 0.910 +0.090 -0.196 | 0.711 +0.289 -0.378 | 0.848 +0.152 -0.248 |
| 21 | 0.816 +0.184 -0.216 | 0.813 +0.187 -0.480 | 0.817 +0.183 -0.245 | 0.899 +0.101 -0.232 | 0.697 +0.303 -0.363 | 0.917 +0.083 -0.167 | 0.729 +0.271 -0.329 | 0.856 +0.144 -0.189 |
| 23 | 0.812 +0.188 -0.212 | 0.812 +0.188 -0.479 | 0.812 +0.188 -0.240 | 0.894 +0.106 -0.227 | 0.685 +0.315 -0.352 | 0.917 +0.083 -0.203 | 0.723 +0.277 -0.323 | 0.853 +0.147 -0.187 |
| 25 | 0.823 +0.177 -0.223 | 0.830 +0.170 -0.496 | 0.820 +0.180 -0.249 | 0.901 +0.099 -0.235 | 0.700 +0.300 -0.366 | 0.925 +0.075 -0.211 | 0.740 +0.260 -0.407 | 0.862 +0.138 -0.195 |
| 27 | 0.819 +0.181 -0.219 | 0.826 +0.174 -0.493 | 0.815 +0.185 -0.315 | 0.897 +0.103 -0.231 | 0.693 +0.307 -0.360 | 0.923 +0.077 -0.173 | 0.735 +0.265 -0.335 | 0.858 +0.142 -0.250 |
| 29 | 0.823 +0.177 -0.223 | 0.841 +0.159 -0.507 | 0.816 +0.184 -0.387 | 0.904 +0.096 -0.237 | 0.703 +0.297 -0.370 | 0.929 +0.071 -0.179 | 0.746 +0.254 -0.346 | 0.860 +0.140 -0.260 |
| 31 | 0.818 +0.182 -0.218 | 0.853 +0.147 -0.519 | 0.803 +0.197 -0.231 | 0.899 +0.101 -0.233 | 0.688 +0.312 -0.301 | 0.934 +0.066 -0.184 | 0.743 +0.257 -0.298 | 0.854 +0.146 -0.239 |
| 33 | 0.824 +0.176 -0.224 | 0.851 +0.149 -0.517 | 0.813 +0.187 -0.241 | 0.897 +0.103 -0.278 | 0.697 +0.303 -0.297 | 0.932 +0.068 -0.182 | 0.749 +0.251 -0.305 | 0.861 +0.139 -0.220 |
| 35 | 0.819 +0.181 -0.219 | 0.852 +0.148 -0.518 | 0.805 +0.195 -0.377 | 0.894 +0.106 -0.275 | 0.688 +0.312 -0.321 | 0.932 +0.068 -0.182 | 0.745 +0.255 -0.345 | 0.856 +0.144 -0.256 |
| 37 | 0.819 +0.181 -0.219 | 0.869 +0.131 -0.202 | 0.798 +0.202 -0.298 | 0.899 +0.101 -0.232 | 0.689 +0.311 -0.289 | 0.939 +0.061 -0.161 | 0.751 +0.249 -0.251 | 0.854 +0.146 -0.247 |
| 39 | 0.817 +0.183 -0.217 | 0.857 +0.143 -0.524 | 0.800 +0.200 -0.371 | 0.896 +0.104 -0.277 | 0.685 +0.315 -0.352 | 0.934 +0.066 -0.184 | 0.745 +0.255 -0.345 | 0.853 +0.147 -0.253 |
| 41 | 0.817 +0.183 -0.217 | 0.855 +0.145 -0.521 | 0.801 +0.199 -0.372 | 0.897 +0.103 -0.278 | 0.686 +0.314 -0.311 | 0.934 +0.066 -0.184 | 0.742 +0.258 -0.342 | 0.854 +0.146 -0.254 |

**Supplemental Table 9**.

The probability values or p-values of the Wilcoxon signed-rank test (two-sided) for the performance metrics of the k-NN classifier by records and by majority votes (PAF as the true class) for the RR, QT intervals with odd values of k ranging from 3 to 41. The displayed metrics are accuracy (ACC), true positive rate (TPR), true negative rate (TNR), area under the receiver operating characteristic curve (AUC), positive predictive value (PPV), negative predictive value (NPV), F_1_ score for PAF subjects (F_1_ PAF) and F_1_ score for control subjects (F_1_ CTR).

| *k* | ACC | TPR | TNR | AUC | PPV | NPV | F_1_ (PAF) | F_1_ (CTR) |
| --- | --- | --- | --- | --- | --- | --- | --- | --- |
| 3 | 2.05e-55 | 3.33e-165 | 3.33e-165 | 1.53e-115 | 3.33e-165 | 2.40e-51 | 3.33e-165 | 1.40e-75 |
| 5 | 3.49e-37 | 3.97e-163 | 1.03e-151 | 4.76e-121 | 6.21e-165 | 2.90e-37 | 1.42e-163 | 7.46e-73 |
| 7 | 2.98e-16 | 3.79e-139 | 2.11e-74 | 3.20e-124 | 3.27e-113 | 4.29e-23 | 1.02e-135 | 1.88e-48 |
| 9 | 1.03e-26 | 6.70e-64 | 2.13e-51 | 9.56e-105 | 1.03e-31 | 6.15e-34 | 1.81e-61 | 2.63e-55 |
| 11 | 1.27e-25 | 1.42e-71 | 6.29e-64 | 2.09e-124 | 3.16e-11 | 3.67e-36 | 3.11e-57 | 3.27e-62 |
| 13 | 2.48e-26 | 1.38e-22 | 4.53e-36 | 6.02e-103 | 1.31e-02 | 6.56e-45 | 1.09e-21 | 2.34e-55 |
| 15 | 2.24e-64 | 9.36e-23 | 1.61e-23 | 3.49e-114 | 1.09e-04 | 4.41e-101 | 2.73e-10 | 8.95e-82 |
| 17 | 1.27e-61 | 5.99e-75 | 5.84e-04 | 6.63e-115 | 6.81e-01 | 1.80e-125 | 1.68e-23 | 3.57e-75 |
| 19 | 1.35e-65 | 8.17e-92 | 5.76e-04 | 6.30e-116 | 4.16e-02 | 4.56e-132 | 4.49e-31 | 8.19e-79 |
| 21 | 4.90e-54 | 4.96e-81 | 1.55e-06 | 8.18e-114 | 5.67e-02 | 7.03e-123 | 1.41e-24 | 6.14e-72 |
| 23 | 5.31e-57 | 7.16e-74 | 6.96e-12 | 3.81e-123 | 1.18e-02 | 2.38e-128 | 3.36e-20 | 1.65e-76 |
| 25 | 3.13e-45 | 1.36e-78 | 1.06e-06 | 2.28e-108 | 5.75e-01 | 2.92e-131 | 2.11e-14 | 1.46e-63 |
| 27 | 4.53e-36 | 3.00e-65 | 4.18e-07 | 6.08e-113 | 7.11e-01 | 9.98e-129 | 2.63e-09 | 7.34e-60 |
| 29 | 3.60e-24 | 1.58e-69 | 1.31e-03 | 3.24e-108 | 8.37e-01 | 1.04e-121 | 4.68e-07 | 3.05e-41 |
| 31 | 1.63e-37 | 2.04e-79 | 2.38e-09 | 2.52e-109 | 2.26e-01 | 2.17e-128 | 1.52e-12 | 1.71e-60 |
| 33 | 6.15e-22 | 3.40e-68 | 1.73e-03 | 3.73e-102 | 1.71e-01 | 1.14e-120 | 4.51e-05 | 1.08e-40 |
| 35 | 1.37e-33 | 1.58e-81 | 6.32e-06 | 2.67e-111 | 8.29e-01 | 9.83e-127 | 6.20e-11 | 4.81e-55 |
| 37 | 4.93e-21 | 2.43e-85 | 3.71e-02 | 3.98e-99 | 9.45e-02 | 3.94e-129 | 7.38e-07 | 1.22e-39 |
| 39 | 3.01e-23 | 1.79e-88 | 2.34e-02 | 1.07e-106 | 2.32e-01 | 1.69e-131 | 4.75e-08 | 2.30e-44 |
| 41 | 1.26e-21 | 7.79e-81 | 1.70e-02 | 1.22e-106 | 2.13e-01 | 2.07e-123 | 1.09e-06 | 6.61e-41 |

**Supplemental Table 10**.

The probability values or p-values of the Wilcoxon signed-rank test (two-sided) for the performance metrics of the k-NN classifier by records and by majority votes (PAF as the true class) for the RR, TQ intervals with odd values of k ranging from 3 to 41. The displayed metrics are accuracy (ACC), true positive rate (TPR), true negative rate (TNR), area under the receiver operating characteristic curve (AUC), positive predictive value (PPV), negative predictive value (NPV), F_1_ score for PAF subjects (F_1_ PAF) and F_1_ score for control subjects (F_1_ CTR).

| *k* | ACC | TPR | TNR | AUC | PPV | NPV | F_1_ (PAF) | F_1_ (CTR) |
| --- | --- | --- | --- | --- | --- | --- | --- | --- |
| 3 | 6.69e-15 | 4.74e-160 | 1.17e-95 | 4.54e-115 | 9.65e-159 | 6.05e-20 | 1.50e-159 | 1.70e-52 |
| 5 | 7.54e-36 | 1.92e-59 | 8.15e-81 | 2.46e-96 | 8.18e-24 | 2.94e-39 | 1.63e-50 | 1.83e-67 |
| 7 | 2.56e-20 | 3.76e-01 | 7.56e-04 | 1.60e-36 | 3.03e-05 | 9.37e-67 | 2.96e-05 | 3.92e-41 |
| 9 | 4.30e-02 | 9.49e-25 | 4.65e-24 | 2.80e-39 | 4.88e-29 | 6.25e-85 | 1.20e-04 | 1.25e-09 |
| 11 | 6.57e-02 | 2.74e-94 | 2.83e-45 | 1.28e-40 | 1.90e-23 | 9.37e-110 | 1.07e-03 | 1.30e-03 |
| 13 | 1.02e-04 | 7.09e-92 | 3.88e-66 | 1.54e-29 | 2.81e-41 | 1.61e-108 | 8.09e-02 | 1.43e-01 |
| 15 | 4.33e-04 | 7.28e-93 | 9.82e-55 | 4.02e-32 | 3.11e-41 | 8.28e-116 | 2.64e-02 | 4.01e-01 |
| 17 | 6.75e-07 | 2.38e-91 | 1.64e-60 | 3.14e-26 | 4.43e-40 | 3.42e-114 | 2.68e-03 | 7.68e-03 |
| 19 | 4.59e-11 | 2.45e-94 | 1.84e-58 | 9.01e-19 | 3.15e-49 | 3.65e-115 | 9.07e-09 | 5.96e-03 |
| 21 | 2.43e-08 | 4.47e-104 | 4.62e-57 | 6.11e-23 | 5.06e-35 | 1.52e-112 | 1.74e-02 | 4.31e-04 |
| 23 | 1.21e-06 | 9.59e-122 | 2.27e-51 | 1.01e-21 | 2.57e-41 | 6.88e-128 | 1.15e-03 | 1.55e-01 |
| 25 | 4.50e-09 | 1.02e-116 | 3.45e-53 | 1.93e-14 | 1.94e-48 | 6.05e-125 | 5.48e-06 | 3.07e-02 |
| 27 | 8.69e-10 | 1.21e-122 | 2.44e-56 | 3.57e-20 | 1.23e-41 | 3.92e-126 | 1.24e-03 | 1.41e-03 |
| 29 | 7.82e-08 | 4.68e-117 | 1.43e-47 | 1.42e-14 | 4.61e-43 | 1.46e-122 | 1.99e-04 | 4.40e-02 |
| 31 | 6.22e-08 | 1.06e-128 | 3.09e-48 | 1.40e-12 | 6.45e-38 | 1.28e-126 | 1.84e-02 | 5.83e-03 |
| 33 | 1.08e-04 | 8.87e-129 | 2.19e-40 | 4.83e-14 | 6.68e-33 | 9.86e-130 | 8.94e-02 | 3.69e-01 |
| 35 | 1.52e-05 | 9.32e-121 | 5.82e-43 | 3.47e-12 | 1.20e-35 | 9.20e-126 | 2.65e-02 | 1.55e-01 |
| 37 | 1.21e-06 | 6.57e-125 | 1.30e-41 | 1.83e-13 | 3.67e-36 | 3.38e-130 | 3.25e-03 | 8.26e-02 |
| 39 | 4.04e-06 | 1.22e-121 | 4.17e-38 | 1.55e-09 | 2.24e-37 | 7.61e-132 | 1.21e-03 | 3.66e-01 |
| 41 | 7.41e-11 | 9.49e-121 | 1.59e-46 | 8.17e-09 | 1.15e-43 | 7.75e-126 | 4.21e-06 | 9.31e-03 |

**Supplemental Table 11**.

The probability values or p-values of the Wilcoxon signed-rank test (two-sided) for the performance metrics of the k-NN classifier by records and by majority votes (PAF as the true class) for the QT, TQ intervals with odd values of k ranging from 3 to 41. The displayed metrics are accuracy (ACC), true positive rate (TPR), true negative rate (TNR), area under the receiver operating characteristic curve (AUC), positive predictive value (PPV), negative predictive value (NPV), F_1_ score for PAF subjects (F_1_ PAF) and F_1_ score for control subjects (F_1_ CTR).

| *k* | ACC | TPR | TNR | AUC | PPV | NPV | F_1_ (PAF) | F_1_ (CTR) |
| --- | --- | --- | --- | --- | --- | --- | --- | --- |
| 3 | 7.70e-55 | 3.33e-165 | 6.31e-164 | 5.57e-93 | 3.33e-165 | 1.16e-48 | 3.33e-165 | 1.30e-75 |
| 5 | 2.73e-27 | 6.05e-164 | 1.11e-152 | 5.85e-74 | 2.66e-164 | 2.90e-26 | 5.45e-164 | 1.09e-62 |
| 7 | 3.04e-13 | 1.44e-145 | 9.70e-59 | 3.96e-64 | 2.28e-130 | 9.83e-27 | 1.09e-145 | 1.18e-48 |
| 9 | 7.95e-21 | 2.93e-71 | 2.92e-46 | 1.04e-86 | 1.50e-31 | 6.22e-34 | 9.89e-68 | 1.32e-52 |
| 11 | 9.22e-16 | 1.27e-52 | 1.41e-42 | 1.78e-92 | 7.70e-10 | 4.33e-28 | 4.41e-46 | 1.02e-44 |
| 13 | 3.06e-26 | 5.83e-11 | 8.75e-28 | 5.61e-89 | 1.49e-01 | 3.16e-51 | 1.34e-13 | 5.53e-56 |
| 15 | 6.85e-33 | 1.52e-21 | 3.50e-03 | 3.04e-90 | 7.63e-02 | 4.64e-89 | 9.56e-03 | 3.01e-51 |
| 17 | 4.55e-46 | 3.14e-82 | 4.61e-01 | 1.22e-103 | 3.02e-01 | 3.24e-123 | 2.03e-21 | 1.64e-58 |
| 19 | 4.09e-45 | 3.30e-94 | 7.34e-01 | 5.43e-115 | 2.64e-01 | 9.43e-132 | 1.32e-20 | 7.08e-62 |
| 21 | 2.13e-41 | 3.58e-103 | 8.10e-01 | 1.02e-119 | 5.59e-01 | 2.05e-136 | 2.10e-20 | 2.13e-57 |
| 23 | 3.02e-31 | 2.43e-73 | 2.89e-01 | 5.73e-106 | 3.57e-02 | 2.60e-125 | 1.17e-08 | 1.68e-50 |
| 25 | 6.58e-25 | 7.08e-80 | 3.46e-01 | 3.02e-114 | 5.43e-02 | 4.21e-127 | 2.30e-06 | 2.26e-47 |
| 27 | 3.81e-14 | 4.25e-67 | 8.83e-01 | 1.60e-122 | 3.57e-02 | 1.86e-115 | 8.67e-04 | 1.06e-30 |
| 29 | 1.97e-16 | 1.41e-74 | 4.96e-01 | 8.18e-124 | 3.89e-02 | 3.10e-121 | 2.05e-04 | 5.85e-33 |
| 31 | 3.17e-17 | 1.77e-69 | 4.76e-01 | 7.01e-119 | 1.62e-02 | 8.80e-124 | 1.56e-03 | 3.62e-36 |
| 33 | 8.97e-19 | 2.67e-80 | 5.71e-02 | 2.42e-117 | 2.19e-01 | 5.75e-129 | 1.54e-04 | 6.14e-40 |
| 35 | 1.23e-19 | 2.85e-83 | 9.74e-02 | 1.42e-111 | 4.83e-02 | 8.65e-129 | 9.68e-05 | 2.44e-40 |
| 37 | 1.08e-20 | 5.25e-89 | 1.09e-01 | 2.04e-108 | 8.30e-03 | 4.29e-130 | 5.50e-06 | 1.93e-40 |
| 39 | 1.40e-26 | 4.99e-93 | 1.10e-03 | 1.17e-99 | 3.14e-01 | 1.74e-133 | 3.53e-08 | 3.88e-48 |
| 41 | 4.34e-22 | 1.42e-102 | 3.30e-02 | 3.61e-104 | 1.72e-01 | 4.44e-132 | 2.57e-08 | 1.51e-41 |

**Supplemental Table 12**.

The probability values or p-values of the Wilcoxon signed-rank test (two-sided) for the performance metrics of the k-NN classifier by records and by majority votes (PAF as the true class) for the RR, QT, TQ intervals with odd values of k ranging from 3 to 41. The displayed metrics are accuracy (ACC), true positive rate (TPR), true negative rate (TNR), area under the receiver operating characteristic curve (AUC), positive predictive value (PPV), negative predictive value (NPV), F_1_ score for PAF subjects (F_1_ PAF) and F_1_ score for control subjects (F_1_ CTR).

| *k* | ACC | TPR | TNR | AUC | PPV | NPV | F_1_ (PAF) | F_1_ (CTR) |
| --- | --- | --- | --- | --- | --- | --- | --- | --- |
| 3 | 1.91e-106 | 3.28e-108 | 2.41e-15 | 2.53e-159 | 4.37e-14 | 4.01e-137 | 7.85e-73 | 1.07e-108 |
| 5 | 1.29e-70 | 5.27e-101 | 6.30e-06 | 1.49e-146 | 2.76e-03 | 3.21e-127 | 2.70e-42 | 1.38e-77 |
| 7 | 8.87e-71 | 1.47e-97 | 4.41e-17 | 6.17e-148 | 1.97e-06 | 1.26e-137 | 2.81e-35 | 7.56e-86 |
| 9 | 9.96e-49 | 2.31e-90 | 2.09e-09 | 9.51e-140 | 1.51e-02 | 3.83e-135 | 4.39e-20 | 8.10e-68 |
| 11 | 1.79e-42 | 1.13e-73 | 2.01e-08 | 2.44e-141 | 6.81e-01 | 3.21e-139 | 4.80e-09 | 2.94e-65 |
| 13 | 5.28e-47 | 1.15e-73 | 1.87e-16 | 2.33e-137 | 2.35e-02 | 5.27e-139 | 5.64e-12 | 3.46e-73 |
| 15 | 2.84e-35 | 1.09e-68 | 6.43e-10 | 2.50e-132 | 1.02e-01 | 1.28e-131 | 1.15e-08 | 4.14e-59 |
| 17 | 2.67e-32 | 2.93e-69 | 2.17e-09 | 2.34e-123 | 1.10e-01 | 1.78e-135 | 4.13e-09 | 1.93e-56 |
| 19 | 1.24e-22 | 2.35e-65 | 5.59e-05 | 2.81e-121 | 6.48e-01 | 3.41e-129 | 6.97e-05 | 1.62e-46 |
| 21 | 6.46e-26 | 5.30e-81 | 3.59e-06 | 1.37e-125 | 5.35e-01 | 7.89e-137 | 7.55e-08 | 8.47e-48 |
| 23 | 4.53e-24 | 2.05e-71 | 4.85e-05 | 4.15e-124 | 6.18e-02 | 7.53e-132 | 3.77e-04 | 2.13e-48 |
| 25 | 5.00e-34 | 9.51e-90 | 1.16e-08 | 1.32e-131 | 6.65e-01 | 2.06e-134 | 1.95e-11 | 8.15e-54 |
| 27 | 7.57e-31 | 3.63e-90 | 2.38e-08 | 3.08e-127 | 8.23e-01 | 2.14e-131 | 3.10e-09 | 6.81e-53 |
| 29 | 3.19e-32 | 5.66e-96 | 1.37e-08 | 7.49e-123 | 1.73e-01 | 6.63e-139 | 6.13e-13 | 7.46e-52 |
| 31 | 1.71e-23 | 3.54e-109 | 3.16e-04 | 5.86e-121 | 6.60e-01 | 2.46e-136 | 1.34e-10 | 9.33e-43 |
| 33 | 2.15e-29 | 8.43e-106 | 1.09e-04 | 1.81e-110 | 9.07e-01 | 3.39e-141 | 6.26e-13 | 5.91e-51 |
| 35 | 1.49e-28 | 2.85e-113 | 1.05e-04 | 1.17e-109 | 8.24e-01 | 2.45e-139 | 9.84e-14 | 4.12e-46 |
| 37 | 6.60e-22 | 5.68e-122 | 2.30e-02 | 1.14e-110 | 4.27e-01 | 7.04e-139 | 1.45e-11 | 1.61e-40 |
| 39 | 4.85e-23 | 8.35e-111 | 4.78e-03 | 7.05e-111 | 6.60e-01 | 9.17e-141 | 3.32e-11 | 2.68e-41 |
| 41 | 2.96e-27 | 9.70e-104 | 6.37e-05 | 1.75e-108 | 5.19e-01 | 9.06e-143 | 6.02e-10 | 5.53e-49 |
