# Supplementary figures and images for: ECG Restitution Analysis and Machine Learning to Detect Paroxysmal Atrial Fibrillation: Insight from the Equine Athlete as a Model for Human Athletes
Source: Function (Oxf). 2020 Nov 18;2(1):zqaa031. doi: 10.1093/function/zqaa031 (PMC8788737; doi:10.1093/function/zqaa031)

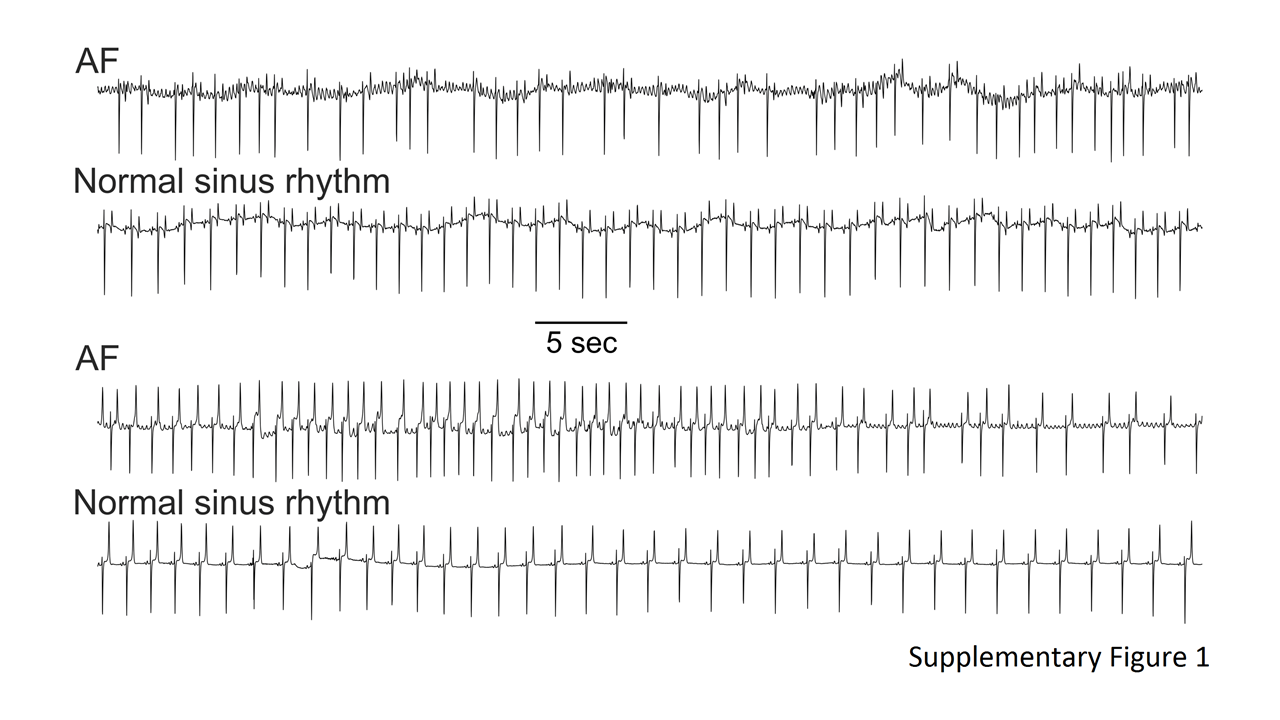

Supplement: zqaa031_Supplementary_Data [file zqaa031_supplementary_data.zip › Supplementary figure 1.tif]

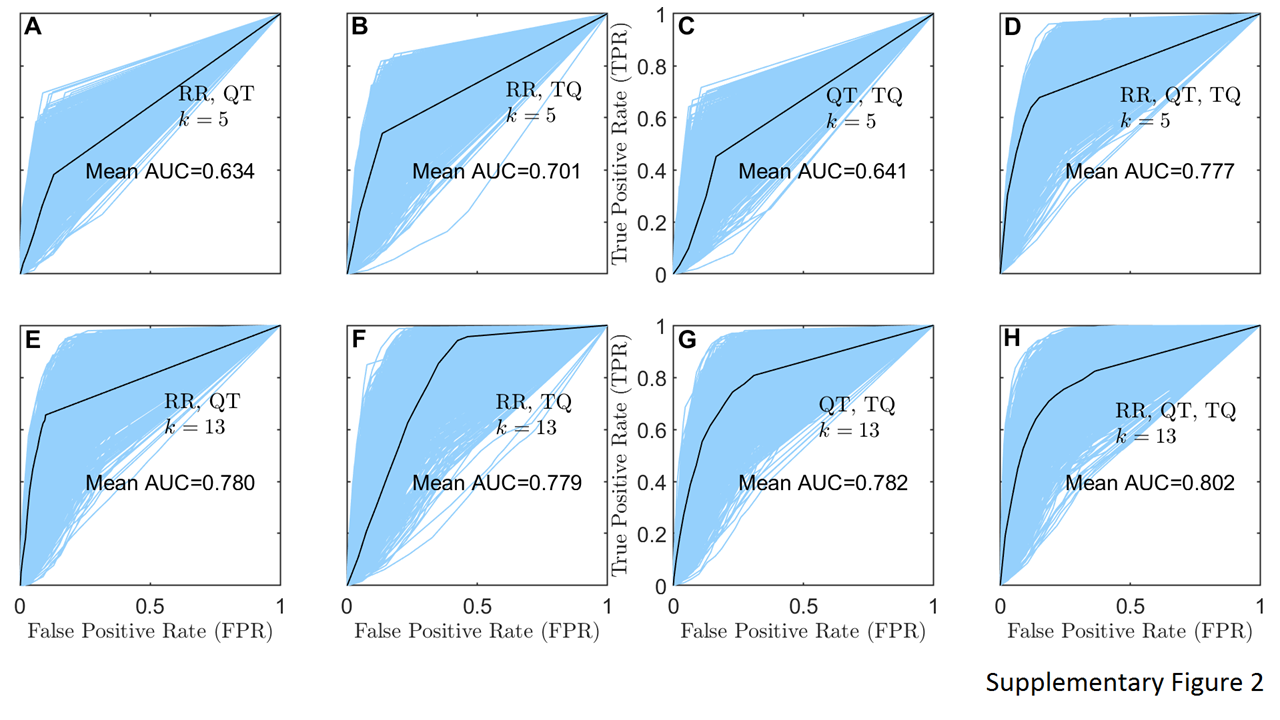

Supplement: zqaa031_Supplementary_Data [file zqaa031_supplementary_data.zip › Supplementary figure 2.tif]

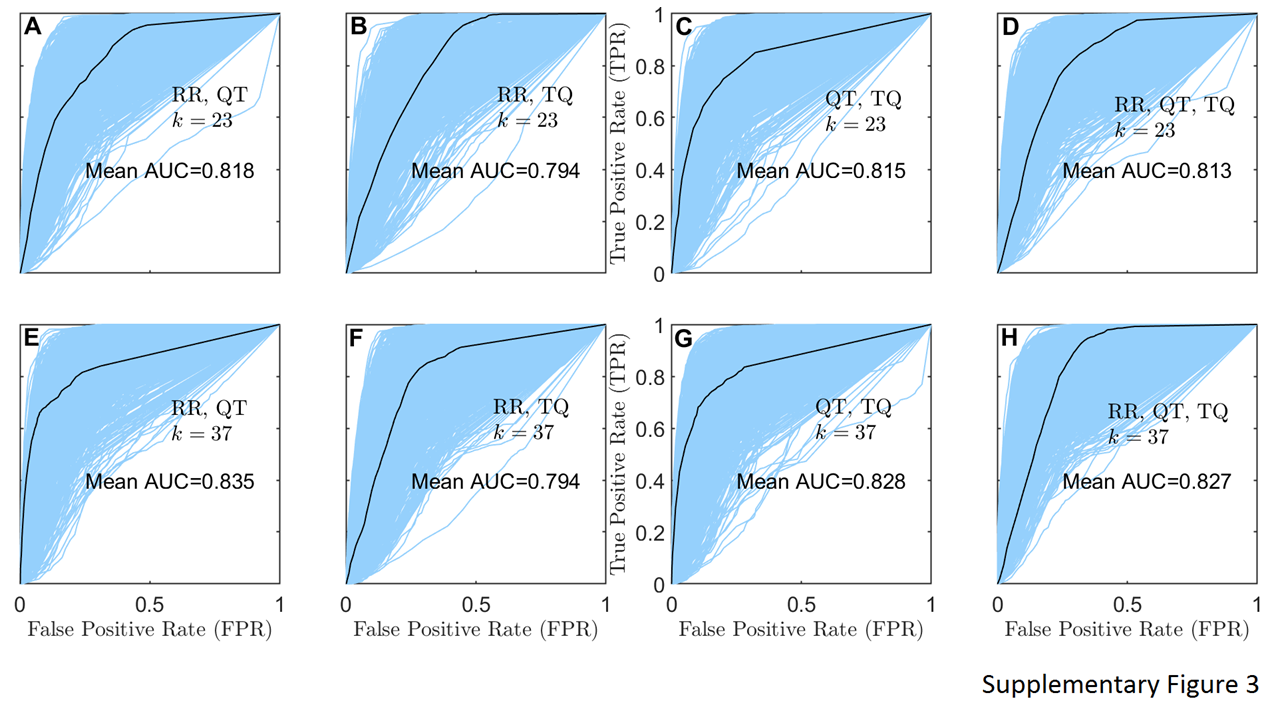

Supplement: zqaa031_Supplementary_Data [file zqaa031_supplementary_data.zip › Supplementary figure 3.tif]

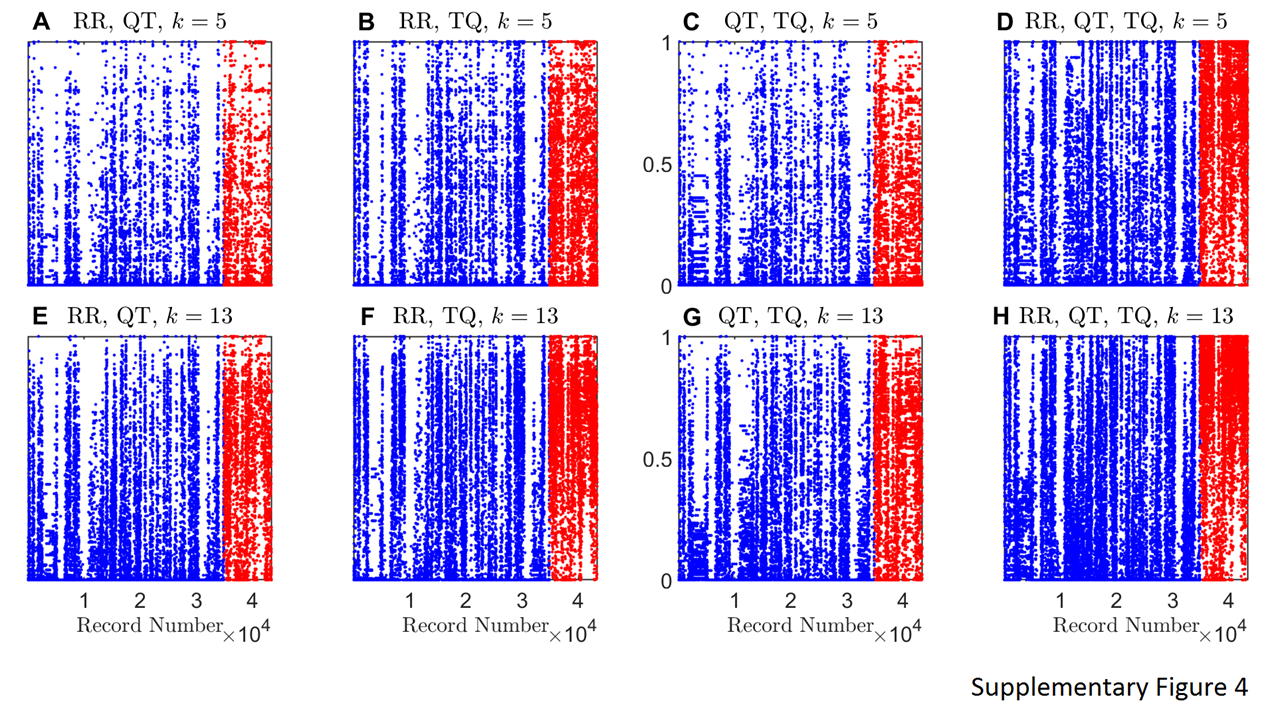

Supplement: zqaa031_Supplementary_Data [file zqaa031_supplementary_data.zip › Supplementary figure 4.tif]

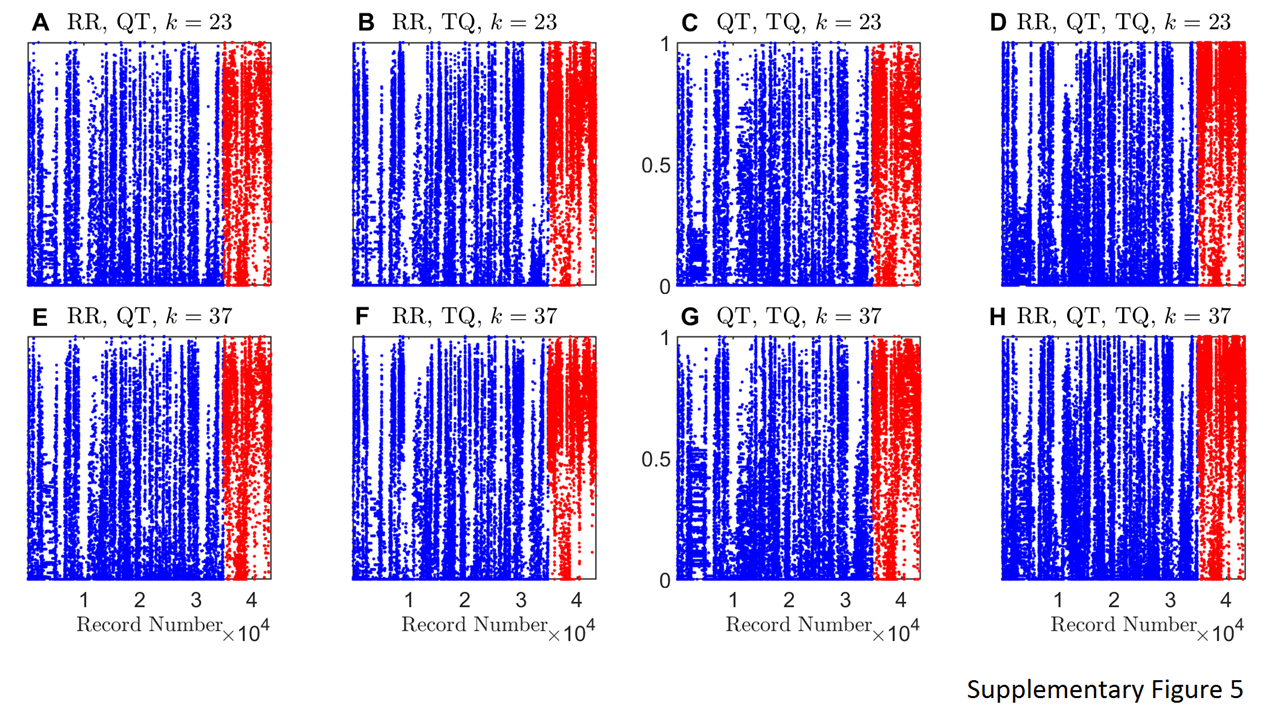

Supplement: zqaa031_Supplementary_Data [file zqaa031_supplementary_data.zip › Supplementary figure 5.tif]
